# Localization of the Priming Factors CAPS1 and CAPS2 in Mouse Sensory Neurons Is Determined by Their N-Termini

**DOI:** 10.3389/fnmol.2022.674243

**Published:** 2022-04-14

**Authors:** Angelina Staudt, Olga Ratai, Aicha Bouzouina, Claudia Fecher-Trost, Ahmed Shaaban, Hawraa Bzeih, Alexander Horn, Ali H. Shaib, Margarete Klose, Veit Flockerzi, Marcel A. Lauterbach, Jens Rettig, Ute Becherer

**Affiliations:** ^1^Department of Cellular Neurophysiology, Center for Integrative Physiology and Molecular Medicine, Saarland University, Homburg, Germany; ^2^Department of Experimental and Clinical Pharmacology and Toxicology, Preclinical Center for Molecular Signaling (PZMS), Saarland University, Homburg, Germany; ^3^Department of Neuroscience, University of Copenhagen, København, Denmark; ^4^Department of Organic Chemistry, Saarland University, Saarbrücken, Germany; ^5^Institute for Neuro- and Sensory Physiology, University Medical Center Göttingen, Göttingen, Germany; ^6^Department of Molecular Imaging, Center for Integrative Physiology and Molecular Medicine, Saarland University, Homburg, Germany

**Keywords:** dorsal root ganglion, cellular localization, intracellular trafficking, synapse, active zone, STED microscopy, FRAP

## Abstract

Both paralogs of the calcium-dependent activator protein for secretion (CAPS) are required for exocytosis of synaptic vesicles (SVs) and large dense core vesicles (LDCVs). Despite approximately 80% sequence identity, CAPS1 and CAPS2 have distinct functions in promoting exocytosis of SVs and LDCVs in dorsal root ganglion (DRG) neurons. However, the molecular mechanisms underlying these differences remain enigmatic. In this study, we applied high- and super-resolution imaging techniques to systematically assess the subcellular localization of CAPS paralogs in DRG neurons deficient in both CAPS1 and CAPS2. CAPS1 was found to be more enriched at the synapses. Using – in-depth sequence analysis, we identified a unique CAPS1 N-terminal sequence, which we introduced into CAPS2. This CAPS1/2 chimera reproduced the pre-synaptic localization of CAPS1 and partially rescued synaptic transmission in neurons devoid of CAPS1 and CAPS2. Using immunoprecipitation combined with mass spectrometry, we identified CAPS1-specific interaction partners that could be responsible for its pre-synaptic enrichment. Taken together, these data suggest an important role of the CAPS1-N terminus in the localization of the protein at pre-synapses.

## Introduction

The calcium-dependent activator protein for secretion (CAPS) is a multi-domain protein involved in Ca^2+^-dependent exocytosis of large dense core vesicles (LDCVs) and synaptic vesicles (SVs) (Walent et al., 1992; Ann et al., 1997; Renden et al., 2001; Jockusch et al., 2007; Speese et al., 2007; Stevens and Rettig, 2009; Shaib et al., 2018). In mammals, the two CAPS paralogs (CAPS1 and CAPS2) are the products of the *CADPS* and *CADPS2* genes and are expressed in a tissue- and developmental-specific manner (Speidel et al., 2003; Sadakata et al., 2006, 2007c). CAPS1 and CAPS2 share approximately 80% amino acid sequence identity and have an identical protein domain structure (Speidel et al., 2003). The N-terminal region consists of a dynactin 1 binding domain (DBD) (Sadakata et al., 2007b) followed by a C2 and a pleckstrin homology (PH) domain (Grishanin et al., 2002; James et al., 2008). The C-terminus contains a Munc13 homology domain 1 (MHD1) (Koch et al., 2000; Pei et al., 2009; Khodthong et al., 2011), and a dense core vesicle (DCV) binding domain (Grishanin et al., 2002).

Deletion of CAPS1 and CAPS2 (CAPS dKO) in adrenal chromaffin cells leads to a strong reduction in LDCV exocytosis, which can be rescued by overexpression of either CAPS1 or CAPS2 (Liu et al., 2008, 2010; Nguyen Truong et al., 2014) suggesting a redundant function of both paralogs in LDCV exocytosis. In the mammalian central nervous system, most neurons express only one CAPS paralog (Speidel et al., 2003; Sadakata et al., 2006). For example, in excitatory hippocampal neurons, CAPS1 is predominantly expressed and its loss reduces spontaneous and evoked SV (Jockusch et al., 2007) and LDCV (Farina et al., 2015; Eckenstaler et al., 2016) exocytosis. In contrast, cerebellar granule neurons and inhibitory hippocampal interneurons predominantly express CAPS2, which is required for LDCV exocytosis, but not for synaptic transmission (Sadakata et al., 2004; Shinoda et al., 2011). In dorsal root ganglion (DRG) neurons, in which glutamatergic synaptic transmission is regulated through the concomitant release of neuropeptides such as CGRP, substance P, VIP, or NPY, the functions of CAPS1 and CAPS2 differ (Shaib et al., 2018). While CAPS1 is expressed in all DRG neurons and mediates synaptic transmission, CAPS2 is primarily expressed in the peptidergic neurons and specifically promotes exocytosis of LDCVs containing neuropeptides. The functional difference between CAPS1 and CAPS2 in these neurons could result from differential subcellular localization of both proteins (Shaib et al., 2018). Deletion of the C-terminus, including the MHD1 and DCV domains, resulted in impaired synaptic localization of CAPS1 in hippocampal neurons (van Keimpema et al., 2017). The effect of this deletion was not studied for CAPS2 localization. However, the high degree of conservation of the C-terminus between both proteins challenges its role in their differential localization.

Herein, we investigate the molecular mechanism underlying the differential subcellular localization of CAPS1 and CAPS2b and evaluate the significance of a unique CAPS1 N-terminal sequence in the localization of the protein by expressing a CAPS1/2 chimera and wild-type (WT) CAPS1 or CAPS2b in DRG neurons of CAPS dKO mice (Jockusch et al., 2007). Using confocal and stimulated emission depletion (STED) microscopy, we found that CAPS1 and the CAPS1/2 chimera, but not CAPS2b, preferentially accumulate at synapses of DRG neurons. These differences in synaptic localization support previous findings that suggest a role for CAPS1, but not for CAPS2, in synaptic transmission. Our results reveal a distinct function of a unique CAPS1 N-terminal sequence in the localization of the protein at pre-synapses.

## Materials and Methods

### Ethical Considerations

Ethical guidelines for the care and use of laboratory animals, issued by the German Government and approved by the Commissions for Institutional Animal Care and Use at Saarland University, Saarland, Germany, were followed. Mice were housed in individually ventilated cages under specific pathogen-free conditions in a 12-h light-dark cycle with constant access to food and water.

### Cloning

pSFV1-mCAPS2b-HA-IRES-eGFP was based on pSFV1-mCAPS2b-IRES-eGFP (Truong et al., 2014) with an insertion of HA tag directly after the CAPS2b C-Terminus. To generate the CAPS1/2 chimera construct, an IDT custom made vector (Integrated DNA Technologies, Leuven, Belgium) was fused in the pSFV1-mCAPS2b-HA-IRES-eGFP plasmid using *Bam*HI and *Psi*I restriction sites. The IDT vector was 805 bp long and corresponded to the entire N-Terminal sequence of CAPS2b containing the CAPS1 sequence from bp 224 to 256. The sequence replaced in the CAPS2b from CAPS1 was 5′-AAG GAG AAG GAA GAG TTG GAG AGG CTG CAG AAG-3′ and resulted in an exchange of the following 11 amino acids GRNEPEL-QLD to KEKEELERLQK. Successful cloning was confirmed via sequencing. Virus particles were produced via established protocols (Ashery et al., 1999).

Full-length murine CAPS1, CAPS2b, or CAPS1/2 chimera were combined with a 14 amino acid flexible linker sequence followed by a HaloTag and cloned into a lentivirus expression vector with a CMV promoter (pRRLsin.cPPT.CMV.WPRE lentiviral transfer vector, Viral Core Facility, Charité Berlin).

The CAPS1-HaloTag plasmid was generated using overlap extension PCR and subsequent restriction enzyme digestion with *Bam*HI and *Nhe*I. A plasmid containing murine CAPS1 was a generous gift from Eckhard Friauf (Kaiserslautern). The protein was amplified using a forward primer that included a *Bam*HI restriction site 5′-ATA TAC GCG GAT CC^ATG CTG GAC CCT TCG TCC AGC-3′ and a reverse primer to add an overhang for the overlap extension PCR 5′-CCG CTT CCG CCG CTC CCA CCG CGC GCC GC^ATC ATC TTC TTC ATC TTC CTC ATC ACT GTC 3′. The HaloTag was amplified from pENTRA4-HaloTag vector (Cat number 29644, Addgene, Teddington, United Kingdom). A forward primer was designed to add the identical overhang sequence as in CAPS1 5′ GGA GCG GCG GAA GCG GCG GTA CCA TCG AT^ATG GCA GAA ATC GGT ACT GGC TTT CCA TTC-3′ and a reverse primer that included a *Nhe*I restriction site 5′-TAT ACT AGC TAG C^TTA GCC GGA AAT CTC GAG CGT CGA CAG-3′.

CAPS-HaloTag Lentivirus constructs were generated by two successive ligation steps in which CAPS2b and the CAPS1/2 chimera were fused to the HaloTag and inserted into the lentivirus expression vector. CAPS2b or CAPS1/2 were excised with *Bam*HI and *Cla*I from the existing Semliki Forest virus plasmids pSFV1-mCAPS2-eGFP and pSFV1-mCAPS1/2-eGFP described above. The HaloTag was amplified from pENTRA4-HaloTag vector via PCR and *Cla*I and *Nhe*I restriction sites were added to the N- and C-termini, respectively. The primer used for the PCR were 5′-A TAT ACC ATC GAT^ ATG GCA GAA ATC GGT ACT GGC TTT CCA TTC-3′ for *Cla*I and 5′-A TAT ACT AGC TAG CGC GGC CGC GGC GGA TCC TTA^GCC GGA AAT CTC GAG-3′ for *Nhe*I. This HaloTag sequence was introduced in pGEM-t vector and amplified in DH5α for *Cla*I methylation. The pGEM-T-Halo plasmid was cut with *Cla*I and *Nhe*I, and the lentiviral expression vector was cut with *Bam*HI and *Nhe*I. After restriction enzyme digestion, CAPS2b or CAPS1/2 were ligated with the HaloTag using the sticky ends from the *Cla*I digestion, followed by ligation with the vector with the sticky ends from the *Bam*HI and *Nhe*I digestion. Successful cloning was confirmed via sequencing.

### Chromaffin Cell Preparation

Adrenal glands were isolated from E18 or E19 CAPS dKO mice of either sex (Jockusch et al., 2007). Cell preparation was performed as previously described (Liu et al., 2008). On day *in vitro* (DIV) 2 or 3, chromaffin cells were infected with Semliki-Forest virus (SFV) pSFV1-mCAPS1/2-HA-IRES-eGFP and pSFV1-mCAPS2b-HA-IRES-eGFP constructs as previously described (Ashery et al., 1999). Electrophysiological recordings were performed 5.5–6.5 h following infection. Each recorded condition represents ≥ 2 independent cultures. Rescued secretion was characterized using membrane capacitance measurements.

For western blotting, chromaffin cells were collected from WT P0-P2 pups and kept for 2 days in culture. Afterward, cells were infected with pSFV1-mCAPS1/2-HA-IRES-eGFP and pSFV1-mCAPS2b-HA-IRES-eGFP for approximately 6 h. The cells were then washed with phosphate-buffered saline (PBS) before harvesting in homogenization buffer (50 mM Tris-Cl, 150 mM NaCl, 250 μM PMSF, 1% Triton X-100, 1 mM deoxycholic acid, 1 mM EDTA, 1 mM DTT, pH 7.4). Cell lysis was performed by short trituration followed by incubation on ice for 1 h. Afterward, the cells were centrifuged for 5 min at 4°C and 15,700 × *g*. The supernatant was transferred to a fresh tube and the protein concentration was measured. A 1× LDS sample buffer (Thermo Fisher Scientific, Waltham, MA, United States, diluted from 4× and containing 10% β-mercaptoethanol) was added to the samples, which were heated for 5 min at 95°C. Protein expression was analyzed by western blotting.

### Patch-Clamp Analysis

Secretion was characterized by capacitance measurements, as described earlier (Shaaban et al., 2019). Similar measurements were performed for all conditions during individual recording sessions. Vesicle fusion was stimulated via photolytic Ca^2+^-uncaging after the infusion of nitrophenyl-EGTA using a patch pipette. Intracellular calcium concentration before and after Ca^2+^ uncaging was estimated using the two ratiometric FURA dyes, fura-4F and furaptra (MagFura, Thermo Fisher Scientific, Waltham, MA, United States). Intracellular calcium was raised to 800–900 nM using high rate (10 Hz) transient illumination alternating between 350 and 380 nm with the Polychrome V (Till Photonics, Kaufbeuren, Germany), followed by a UV flash for the full uncaging of calcium (JML-C2, Rapp OptoElectronic, Wedel, Germany). The intracellular solution for the Ca^2+^ uncaging experiments was composed of 100 mM Cs-glutamate, 32 mM Cs-HEPES, 8 mM NaCl, 4 mM CaCl_2_, 2 mM Mg-ATP, 0.3 mM GTP, 5 mM Nitrophenyl-EGTA, 0.4 mM fura 4F, 0.4 mM furaptra, and 1 mM Vitamin C, pH 7.2 (osmolarity was adjusted to 290 mOsm). Experiments were performed at room temperature (20 ± 2°C).

### Co-culture of Dorsal Root Ganglion and Spinal Cord Neurons

We investigated CAPS localization using a co-culture system since DRG neurons do not form synapses with each other (Ransom et al., 1977a,b), but generate functional synapses with their natural target cells, the spinal cord (SC) dorsal horn neurons (Gu and MacDermott, 1997; Joseph et al., 2010; Shaib et al., 2018). First DRG neuron cultures from young adult (1–4 weeks old) WT, CAPS2 KO, CAPS1^+/–^ CAPS2^–/–^ and synaptobrevin 2-mRFP KI mice of either sex were made as previously described (Bost et al., 2017). Embryonic DRG neuron cultures were generated from E17-E19 CAPS1 KO, CAPS dKO, and WT mice of either sex (Shaib et al., 2018). The CAPS1 KO, CAPS2 KO, and CAPS dKO genotypes were verified using PCR as previously described (Speidel et al., 2005; Jockusch et al., 2007). Lentivirus infection (see below) was performed on DIV1. The following day, the lentivirus was removed by washing before adding the second order SC interneurons (SC neurons) to the culture. This transduction protocol ensured exclusive transfection of DRG neurons. SC neurons were prepared from WT P0-P2 pups of either sex as previously described (Shaib et al., 2018). DRG/SC co-cultures were maintained in Neurobasal A (NBA) medium (Thermo Fisher Scientific, Waltham, MA, United States) supplemented with fetal calf serum (5% v/v), penicillin and streptomycin (0.2% each), B27 supplement (2%), GlutaMAX (1%, all Thermo Fisher Scientific, Waltham, MA, United States), and human beta-nerve growth factor (0.2 μg/mL, Alomone Labs, Jerusalem, Israel) at 37°C and 5% CO_2_.

### Lentivirus Production and Transduction of Dorsal Root Ganglion Neurons

Lentivirus was produced by transfecting human embryonic kidney 293 FT cells with plasmids encoding for the structural elements, envelope, and transfer genes. Briefly, 80–90% confluent cells cultured in 150 cm^2^ flasks were starved with incomplete DMEM medium for 6 h prior to calcium phosphate transfection. Subsequently, a solution of 1.8 mL water, the lentiviral DNA, and 200 μL of freshly prepared calcium chloride (2.5 M) was prepared, dropped in a 2 mL hank’s balanced salt solution, and incubated for 30 min before transfection of a maximum of two cell culture flasks. The DNA mixture for third-generation lentivirus production (of all CAPS constructs) contained 180 μg DNA of the vector and 80 μg of the helper plasmids pMDLg/pRRE, pRSV-REV, and pMD2.G. The cells were incubated for 6 h before the medium was replaced with supplemented DMEM (100 mM sodium pyruvate, 10% FCS, and 1% NEAA, all Thermo Fisher Scientific, Waltham, MA, United States). After 36 h, the cell culture supernatant was collected, and cell debris was removed using filtration. For ultracentrifugation, the elution was transferred into 35 mL thin-wall polypropylene tubes prefilled with 1.5 mL of 20% sucrose. The virus was centrifuged at 86,500 × *g* for 2.5 h. The virus pellet was resuspended in Dulbecco’s phosphate-buffered saline. Aliquots were flash frozen in liquid nitrogen and stored at −80°C until use.

### Halo Ligand Chloralkane-ATTO590 Synthesis and Staining

Chloralkane-ATTO590 (CA-ATTO590) was synthesized as follows. DIPEA (9.97 μL, 57.1 μmol, 10 equiv.) and a solution of amine SI-1 (1.4 mg, 6.28 μmol 1.1 equiv) in dry DMSO (200 μL) were added to a solution of ATTO590-NHS (90%, 5.0 mg, 5.71 μmol, 1.0 equiv) in dry DMSO (200 μL). After stirring in the dark for 14 h the mixture was diluted with EtOAc (10 mL) and washed with aq. NaHCO_3_ (sat.) and brine. The organic layer was dried (Na_2_SO_4_), and the solvent was removed in a vacuum centrifuge. The crude product was purified by flash chromatography (silica, DCM/MeOH 97:3 to 90:10) and lyophilized to obtain the dye CA-ATTO 590 (4.1 mg, 4.87 μmol, 85%) as a blue to purple solid. TLC (silica, DCM/MeOH 95:5) *R*_*f*_ = 0.12; LCMS (Phenomenex Luna C18, H_2_O/MeCN 9:1 to 100% MeCN, 20 min) *t*_*R*_ = 11.04 min (M + H 796.55).

Cell labeling was performed on DIV6 by replacing the old culture medium with fresh culture medium containing CA-ATTO590 (0.5 μM for confocal or 2.5 μM for STED microscopy). After a 30-min incubation, cells were rinsed twice with NBA medium and incubated in half fresh and half-old NBA culture medium for 3 days before they were used for immunocytochemistry. For the FRAP experiments the staining was performed on DIV8 with the HaloTag ligand TMR (Promega, Madison, WI, United States) at a concentration of 5 μM and with an incubation time of 1 H at 37°C.

### Immunocytochemistry and Confocal Microscopy

The neurons were fixed at room temperature (20 ± 2°C) with 4% paraformaldehyde in PBS (pH 7.4) for 15 min and permeabilized with 0.1% Triton X-100 and 5% normal goat serum in PBS for 30 min. The samples were blocked with 5% normal goat serum in PBS for 10 min and then incubated with the specified primary antibodies (Table 1). After several washing steps, the samples were incubated with secondary antibodies (Table 1) for 45 min at room temperature (20 ± 2°C). For immunostaining containing two primary antibodies raised in the same species, an intermediate blocking step with Fab fragments (Rockland) was performed for 1 h, followed by extended washing steps. The samples were mounted with home-made Mowiol-based mounting medium and imaged with an LSM 780 confocal laser scanning microscope (Carl Zeiss, Oberkochen, Germany) using a 63×/1.4NA oil immersion objective.

**TABLE 1:** Details of antibodies used in this study.

Antibody	Host	Immunogen	Manufacturer and catalog no.	Working dilution
**Primary antibody**				
**Bassoon**	Mouse	Recombinant protein (from rat Bassoon)	Enzo ADI-VAM-PS003	1:400 (ICC)
**Beta-actin**	Mouse	Monoclonal, clone AC-15	Merck A1978	1:10,000 (WB)
**CAPS1**	Rabbit	Recombinant protein (aa 18–107 from mouse CAPS1)	Synaptic Systems 262013	5 μg ∼ 1:50 (IP) 1:1000 (WB) 1:500 (ICC)
**CAPS2**	Rabbit	Recombinant protein (aa 15–89 from mouse CAPS2)	Synaptic Systems 262103	5 μg ∼ 1:50 (IP)
**CAPS2**	Rabbit	Full length CAPS2e -(purified against a CAPS2 specific sequence GSGGGAARPV)	Provided by M. Jung	1:1000 (WB)
**HA**	Rat	Monoclonal antibody (clone 3F10, aa 98–106 from the human influenza hemagglutinin protein)	Roche	1:1000 (WB)
**Homer1**	Rabbit	Recombinant protein (aa 1–196 from human Homer1)	Synaptic Systems 160003	1:1000 (ICC)
**Munc13-1**	Rabbit	Recombinant protein (aa 3–317 from rat Munc13-1)	Synaptic Systems 126103	1:500 (ICC)
**Normal IgG**	Rabbit	N/A	Merck 12-370	5 μg ∼ 1:50 (IP)
**Synapsin1/2**	Guinea Pig	Synthetic peptide (aa 2–28 from rat Synapsin1)	Synaptic Systems 106004	1:1000 (ICC)
**Synaptotagmin1**	Rabbit	Synthetic peptide (aa 1–8 from mouse Synaptotagmin1)	Synaptic Systems 105 103C2	1:75 (live cell for FRAP)
**Secondary antibody**				
**Alexa 488**	Goat	Rabbit	Thermo Fisher Scientific A-11034	1:1000
**Alexa 647**	Goat	Rabbit	Thermo Fisher Scientific A-21245	1:1000
**Alexa 405**	Goat	Mouse	Thermo Fisher Scientific A-31553	1:500 to 1:1000
**FAB fragments**	Goat	Rabbit	Rockland 811-1102	1:50
**IgG F(ab)2 HRP**	Goat	Rabbit	Merck AQ132P	1:5000
**IgG (H + L) HRP**	Goat	Mouse	Thermo Fisher Scientific 32430	1:1000
**IgG HRP**	Goat	Rat	GE Healthcare NA935	1:5000
**STAR RED**	Goat	Mouse	Abberior	1:100
**STAR 580**	Goat	Rabbit	Abberior	1:100
**STAR 580**	Goat	Guinea Pig	Abberior	1:100

The vital immunostaining of active synapses with an anti-body directed against the luminal domain of synaptotagmin1 coupled to Sulfo-Cyanine 2 (syt1-C2) was performed as follows. 9 DIV old DRG-SC co-cultures stained with the HaloTag ligand TMR were incubated for 1 H in a depolarizing solution containing the anti-syt1-C2 anti-body (Table 1) at a dilution of 1:75. Then the cells were washed quickly 1 time with fresh NBA medium and placed back in its old culture medium for 1 to 2 H in the incubator prior to the experiment. The depolarizing solution consisted of the culture medium mixed with a solution containing in mM: 114 KCl, 2 CaCl_2_, 1 MgCl_2_, 20 glucose and 10 HEPES, to obtain a final concentration of 35 mM KCl. This solution induced a mild depolarization of the neurons sufficient to induce synaptic transmission and subsequent endocytosis of SV in order to allow uptake of the anti-body and label active synapses (Truckenbrodt et al., 2018).

### Stimulated Emission Depletion Microscopy

Secondary antibodies used for STED microscopy are listed in Table 1. Samples were mounted with Abberior Mount Solid (Abberior, Göttingen, Germany). Imaging was performed with a four-color STED QuadScan (Abberior, Göttingen, Germany) using 485 nm/0.85 mW, 561 nm/2 mW, and 640 nm/12 mW excitation pulsed lasers, and a 775 nm/1.25 W STED laser. The pinhole size was set to 80 μm (1.0 arbitrary unit) and the probes were visualized with a 100×, NA 1.4 objective (UPLSAPO100XO, Olympus, Hamburg, Germany). The following acquisition protocol was used. First, a single confocal section was recorded at 488, 561, and 640 nm to visualize synapsin, CAPS, and bassoon staining. Then, one central section of a DRG-SC neuron synapse identified by triple labeling was acquired in 2DSTED mode at 561 and 647 nm to visualize the CAPS and bassoon staining. Finally, a stack of about 1 to 1.5 μm depth was recorded of the same synapse in the 3DSTED mode. For the confocal images, the laser power was 12% at 485 nm and 15% for 561 and 640 nm. The pixel size was 80 × 80 nm. For 2DSTED images, the laser power was 25 and 15% at 561 and 640 nm, respectively. The STED laser emitted 25% of the maximal power of 1250 mW (corresponding to 37.5–42.5 mW in the focus, with a repetition rate of 40 MHz) with a gating of 938 ps. The pixel size was 20 × 20 nm. For 3DSTED images, laser powers and STED laser gating were identical to 2DSTED imaging. However, the STED laser emitted at 40% (corresponding to 60–68 mW in the focus, with a repetition rate of 40 MHz). The voxel size was 40 × 40 × 40 nm. The settings used to acquire the STED images with synapsin or the active zone proteins Munc13-1 and Homer1 together with bassoon were similar to those with CAPS, aside from the laser power that had to be adjusted for the individual staining.

The acquired images were deconvolved with Matlab (Mathwork, Natick, MA, United States) prior to analysis. We applied a linear deconvolution method (Wiener filter) using theoretical point spread functions (PSF) and user-adjusted regularization parameters. For the 3D stacks, each plane was deconvolved individually using 2D deconvolution. The confocal PSF was modeled as a 2D Gaussian function, the STED PSF as a 2D Lorentzian function.

### Fluorescence Recovery After Photobleaching

The fluorescence recovery after photobleaching (FRAP) experiment were performed with DRG neurons from mice that were heterozygote for CAPS1 and KO for CAPS2 (CAPS1^+/–^ CAPS2^–/–^). These neurons express CAPS1 albeit at a reduced level in comparison to WT neurons (Speidel et al., 2003). Cells were over expressing either CAPS1-, CAPS2b- or CAPS1/2-Halo chimera and they were stained with the HaloTag ligand TMR and the anti-syt1-C2 antibody. The FRAP experiments were performed with the STED microscope in confocal modus using the 40×/1.4NA oil objective (UPLXAPO40XO, Olympus, Hamburg, Germany). First an overview image was acquired at 488 and 561 nm to identify synapses through the co-localization of TRM and syt1-C2 in small structures along neurites. For the actual FRAP acquisition, 10 images of the identified synapse were recorded at 3 Hz using the 561 nm laser at 10% of its maximal power with a scanning speed of 5 μs per pixel and line accumulation set to 2. Pixel size was 80 nm^2^. For the bleaching step, a central region of the synapse – about 0.8 μm^2^ large – was illuminated twice with the laser at full power using a pixel size of 20 nm^2^ scanning speed of 100 μs per pixel and 8-line accumulations. This step lasted approximatively 6 s and resulted in the complete bleaching of the entire synapse. Then recording of the synapse was resumed immediately as before bleaching for another 120 s to measure the fluorescence recovery. The pinhole was set to 3.13 AU in order to get a thick z-sectioning and avoid z-drifting artifacts.

### Synaptic Transmission Imaging

The imaging setup was described previously (Bost et al., 2017; Shaib et al., 2018). Briefly, an Olympus IX70 microscope (Olympus, Hamburg, Germany) was equipped with a 100x/1.45 NA Plan Apochromat Olympus objective, a TILL-total internal reflection fluorescence (TILL-TIRF) condenser (TILL Photonics, Kaufbeuren, Germany), and a Prime 95 B scientific CMOS camera (Teledyne Photometrics, Tucson, AZ, United States). The final pixel size was 110 nm. For data shown in the Supplementary Material, the QuantEM 512SC camera (Photometrics) was used with a final pixel size of 160 nm. A multi-band argon laser (Spectra-Physics, Stahnsdorf, Germany) emitting at 488 nm was used to excite Synaptophysin-pHluorin (SypHy) fluorescence, and a solid-state laser 85 YCA emitting at 561 nm (Melles Griot Laser Group, Carlsbad, CA, United States) was used to excite CA-ATTO590. A dual-view camera splitter (Visitron Systems, Puchheim, Germany) was applied to separate the red (CAPS-Halo–CA-ATTO590) and green (SypHy) channels.

Secretion was evoked by electrical stimulation via a bipolar platinum-iridium field electrode (#PI2ST30.5B10, MicroProbes, Gaithersburg, MD, United States) and a pulse stimulator (Isolated Pulse Stimulator Model 2100, A-M Systems, Sequim, WA, United States). The measurement protocol was 30 s without stimulus followed by a biphasic 1 ms long 4 V stimulus train at 10 Hz for 30 s to elicit exocytosis of SVs. At the end of the measurement, NH_4_Cl was applied to visualize the entire SV pool. During the measurement, the temperature was maintained at 32°C by a perfusion system with an inline solution heater (Warner Instruments, Holliston, MA, United States). The extracellular solution contained 147 mM NaCl, 2.4 mM KCl, 2.5 mM CaCl2, 1.2 mM MgCl2, 10 mM HEPES, and 10 mM glucose (pH 7.4; 300 mOsm). The NH_4_Cl solution had the same composition as the extracellular solution, but 40 mM NaCl was replaced with equal amount of NH_4_Cl.

### Confocal Image Analysis

Confocal data were analyzed with ImageJ (developed at the National Institutes of Health) on background subtracted single-plane images. We analyzed the co-localization of anti-CAPS1, anti-Munc13-1 antibody, and synaptobrevin 2-mRFP in two different manners: (1) line profile analysis was performed on heterotypic synapses, which were identified by the co-localization of all three proteins with bassoon. The profiles were determined on three pixel-wide lines that were approximately 2 μm long, crossing the synapse along the neurite. The center of the synapse was defined by the Syb2-mRFP maximum fluorescence intensity. This Syb2-mRFP peak was used to register the individual CAPS1 and Munc13-1 curves of each synapse before averaging. (2) Manders’ overlap coefficient for co-localization (Manders et al., 1993) was measured using the JACoP ImageJ plugin (Bolte and Cordelières, 2006) on isolated neurites of DRG neurons. We limited the analysis to neurites that were at least 10 μm long and that contained synaptobrevin 2-mRFP puncta, indicating the presence of heterotypic synapses. In addition, co-localization of CAPS1 with Munc13-1 was illustrated by measuring the fluorescence intensity along a three-pixel wide line within the displayed neurite.

To analyze the expression levels of CAPS1-, CAPS2b-, and CAPS1/2 chimera-Halo, we measured the mean fluorescence intensity for CA-ATTO590 and CAPS antibody signals in a region of interest (ROI) delimiting the cell soma. The distribution of the proteins from the cell soma into its neurite was analyzed by measuring the fluorescence intensity along a three-pixel wide line with a length of 15 μm starting in the cell soma and ending in the neurite. The ratio of the average fluorescence intensities over a distance of 2.5 μm at the beginning and end of the line was calculated.

To analyze the enrichment of CAPS1-, CAPS2b-, and CAPS1/2 chimera-Halo at synapses, we measured the mean fluorescence intensity of an ROI drawn precisely over the synapse and normalized it to the mean fluorescence intensity of an ROI placed on the neurite adjacent to the synapse. Furthermore, we measured the co-localization of CAPS1, CAPS2b, or CAPS1/2 chimera to synapsin using Pearson’s correlation and Manders’ overlap coefficients with the JACoP plugin in ImageJ. This co-localization analysis was limited to synapse rich regions of DRG neurites.

### 3DSTED Image Analysis

The subsynaptic localization of CAPS-Halo proteins was analyzed in comparison to the active zone protein bassoon on the full 3D volume of the synapse using a cluster analysis approach in Imaris (Oxford Instruments, Abingdon, United Kingdom) on the deconvolved images. Initially, protein clusters in each channel (CAPS and bassoon) were identified by the ImarisCell module using the creation wizard with the following settings: background subtraction width 1.6 μm, smoothing filter 0.04 μm, near automatic threshold, split by seed points (0.2 μm). The other settings were adjusted for each image individually. This module provided us with the XYZ center of mass position of the clusters their fluorescence intensity and their volume. Then, we exported the ImarisCells to ImarisSurface, which allowed us to measure the overlap between CAPS and bassoon clusters using the Surface-Surface co-localization Imaris XTension. The position of the center of mass of the two protein clusters allowed us to measure the distance between bassoon and CAPS. Since most 3D-STED images contained more than one active zone defined by a bassoon cluster, we performed a nearest neighbor analysis between the clusters of both proteins. The maximum distance between them was set to 0.3 μm. This nearest-neighbor analysis allowed us to define pairs between a bassoon and a CAPS cluster, which we validated visually. Individual pairs were selected to perform the overlap analysis. We repeated this analysis with anti-Munc13-1, anti-synapsin, and anti-Homer1 antibodies.

### Analysis of Fluorescence Recovery After Photobleaching Experiments

Synaptic fluorescence over time was measured using ImageJ. Data of each synapse were normalized to its average fluorescence prior bleaching. Due to slight time shifts during recording, the data obtained for each synapse was linearly interpolated to generate a fluorescence recovery curve with a time base of 3.3 Hz before averaging the results of all synapses. T1_/2 maximum recovery_ and the immobile fraction was then calculated using the NeuroMatic macro in Igor (WaveMetrics, Portland, OR, United States). Because the recovery was not completed for all synapses after 120 s, we fitted the individual curves with a double exponential function and extended the time line to 500 s. The asymptotic value of y at x infinity (y0) was then taken as the maximum recovery, i.e., the mobile fraction of CAPS-Halo. The immobile fraction was calculated as the inverse of the mobile fraction. Finally, *t*_1/2 maximum recovery_ was calculated by NeuroMatic on the fitted curves.

### Analysis of Synaptic Transmission Imaging

Data were analyzed using ImageJ. Synapses were identified as immobile stained points that reacted to a 40 mM NH_4_Cl application with a strong increase in fluorescence intensity. Synapses were selected by ROIs and the mean gray value was measured as a function of time after background subtraction. The level of expression of SypHy varied between cells, resulting in a different amount of SypHy per SV, which influenced the extent to which the fluorescence intensity changed during stimulation. To overcome this problem, we neutralized all SVs by applying 40 mM NH_4_Cl for 10 s at the end of each experiment, allowing visualization of the entire SV pool at each synapse (Sankaranarayanan and Ryan, 2000). The maximum fluorescence intensity at NH_4_Cl application was then used to normalize the SypHy signal. To homogenize for SypHy expression level, we included in the analysis only synapses with a normalized fluorescence intensity before the stimulus between 0.2 and 0.6. Finally, we quantified the maximal change in fluorescence before and during the stimulus of all selected synapses.

### Immunoprecipitation

Whole mouse brain lysates were prepared for CAPS1 while cerebellum lysates for CAPS2 according to CAPS1 and CAPS2 tissue specific distribution (Speidel et al., 2003; Sadakata et al., 2007c). The tissue was homogenized in lysis buffer [20 mM HEPES, 150 mM KCl, 0.05% (v/v) Tween-20, 100 nM CaCl_2_, 10 μM E64, 2 mM Pefabloc SC, 1 μg/mL Pepstatin A, pH 7.4] containing 300 mM sucrose using a glass potter followed by incubation at 4°C for 45 min. Homogenates were centrifuged twice for 5 min at 4°C and 400 × *g*. The supernatant was transferred into a fresh tube and the protein concentration was measured. The freshly prepared protein lysates were precleared for 1 h at room temperature (20 ± 2°C) with Protein G Dynabeads (Thermo Fisher Scientific, Waltham, MA, United States), previously washed in lysis buffer. Afterward, precleared lysates were incubated with a rabbit anti-CAPS1 antibody (Table 1) or a rabbit anti-CAPS2 antibody (Table 1) for 3 h at room temperature (20 ± 2°C). Incubation of precleared lysates with a rabbit anti-IgG antibody (Table 1) was used as a negative control. Prewashed Protein G Dynabeads were added to the sample and incubated for 1 h or overnight (for CAPS1 or CAPS2, respectively) at room temperature (20 ± 2°C). After incubation, the supernatant was collected, beads were washed, and the antibody protein complex was eluted in 1 × LDS sample buffer. Protein detection was analyzed by western blot and mass spectrometry (MS).

### Western Blotting

Samples were loaded onto SDS-polyacrylamide gradient gels (3–8%). After electrophoresis, proteins were detected using the Pierce Silver Stain Kit (Thermo Fisher Scientific, Waltham, MA, United States) according to the manufacturer’s protocol or were transferred to a nitrocellulose membrane for detection by western blotting. Membranes were blocked with 7% milk in Tris-buffered saline with 0.05% (v/v) Tween-20 (TBST) for 1 h at room temperature (20 ± 2°C). Primary antibodies (Table 1), diluted in TBST with 3.5% milk, were incubated for 1 h at room temperature (20 ± 2°C) or overnight at 4°C. After washing, primary antibodies were detected by incubation with horseradish peroxidase–conjugated secondary antibodies (Table 1) for 1 h at room temperature (20 ± 2°C) in TBST with 3.5% milk. Blots were developed by the Pierce enhanced chemiluminescence western blotting substrate (Thermo Fisher Scientific, Waltham, MA, United States) and imaged using a FluorChem M system (Protein Simple, San Jose, CA, United States).

### Gel Electrophoresis of Proteins and Sample Preparation for Mass Spectrometry

Protein eluates were separated on NuPAGE 4–12% Bis-Tris gradient gels, fixed in 40% ethanol and 10% acetic acid, incubated 3 times for 10 min with water, and stained with Coomassie [0.12% (w/v) Coomassie G-250 (20% (v/v) methanol, 10% (v/v) phosphoric acid, and 10% (w/v) ammonium sulfate]. Stained gel areas were cut into three pieces and washed twice alternating between buffer A (50 mM NH_4_HCO_3_) and buffer B [50 mM NH_4_HCO_3_/50% (v/v) acetonitrile]. The reduction of disulfide bonds was obtained by incubation at 56°C for 30 min in 10 mM dithiothreitol in buffer A, followed by carbamidomethylation at 21°C in darkness for 30 min in 5 mM iodoacetamide in buffer A. Afterward, the gel pieces were washed twice alternating between buffer A and B and then dried in a vacuum centrifuge. For in-gel digestion, the gel pieces were incubated in the presence of 20 μL of porcine trypsin (10 ng/μL, Promega) at 37°C overnight. Tryptic peptides were extracted twice with 50 μL of extraction buffer (2.5% formic acid/50% acetonitrile) in an ultrasonic bath. Extracted peptides were combined and concentrated in a vacuum centrifuge and resuspended in 21 μL of 0.1% formic acid.

### Nano ESI-LC-MS^2^ Measurements

Tryptic peptide extracts (6 μL) were analyzed by nanoflow LC-HR-MS/MS (Ultimate 3000 RSLC nano UHPLC-system coupled to an LTQ Orbitrap Velos Pro, all Thermo Fisher Scientific, Waltham, MA, United States). Peptides were trapped on a trap column (100 μm × 2 cm, Acclaim PepMap100C18, 5 μm, Thermo Fisher Scientific, Waltham, MA, United States) and separated on a reversed phase column (Acclaim PepMap capillary column, C18; 2 μm; 75 μm × 25 cm, Thermo Fisher Scientific, Waltham, MA, United States) at a flow rate of 200 nL/min for 120 min in a gradient with buffer A (water and 0.1% formic acid) and B (90% acetonitrile and 0.1% formic acid). Eluted peptides were directly sprayed into the mass spectrometer through a coated silica electrospray emitter (PicoTipEmitter, 30 μm, New Objective, Littleton, MA, United States) and ionized at 2.2 kV. MS spectra were acquired in a data-dependent mode (automatic switch between full scan MS and MS^2^). Full scan MS spectra (m/z 300–1,700) were acquired in the Orbitrap analyzer using a target value of 10^6^. The 10 most intense peptide ions with charge states > + 2 were fragmented in the high-pressure linear ion trap by low-energy collision-induced dissociation (35% normalized collision energy).

### Raw LC-MS^2^ Data Analysis

Tryptic peptides were identified using the MASCOT algorithm (Matrix Science, Boston, MA, United States) and TF Proteome Discoverer 1.4 (Thermo Fisher Scientific, Waltham, MA, United States). Peptides were matched to tandem mass spectra by Mascot version 2.4.0 (Matrix Science, Boston, MA, United States) by searching the SwissProt database (version 2018_05, number of protein sequences 557.992 containing 16.992 *Mus musculus* sequences) against mouse proteins. MS^2^ spectra were matched with a mass tolerance of 7 ppm for precursor masses and 0.5 Da for peptide fragment ions. We used tryptic digestion and allowed up to two missed cleavage sites. Cysteine carbamidomethylation was set as a fixed modification and deamidation of asparagine and glutamine, acetylation of lysine, and oxidation of methionine were set as variable modifications. MASCOT output files (.dat) were loaded into Scaffold (Version 4.8.8, Proteome Software Inc., Portland, OR, United States). To ensure significant protein identification, the protein probability filter was set to 95% and the peptide probability filter was set to 90% (protein FDR: 0.6 or 0.0% decoy, peptide FDR: 0.1 or 0.0% decoy for CAPS1 or CAPS2, respectively). The identification of two unique peptides per protein was set as the minimum for protein identification. Protein probabilities were assigned by the Protein Prophet algorithm (Nesvizhskii et al., 2003). Proteins that contained similar peptides and could not be differentiated based on MS/MS analysis alone were grouped to satisfy the principles of parsimony.

### Statistical Analyses

In the box plots, the boundaries of the box represent the 25th to 75th percentile, a black line or a white dot within the box marks the median, while the white line corresponds to the mean. Whiskers above and below the box indicate the 90th and 10th percentiles, respectively. In all other plots, error bars represent the standard error of the mean. Significance was tested by one-way ANOVA with Tukey’s *post hoc* test when the data followed a normal distribution. Otherwise, ANOVA on ranks with Dunn’s *post hoc* test was applied. A *p*-value < 0.05 was considered significant. N is the number of cultures, while n is the number of measured neurons unless specified otherwise. Statistical tests were performed with Sigma Plot 13 (Systat Software, Erkrath, Germany). Graphs were generated by Sigma Plot 13 (Systat Software, Erkrath, Germany), Igor (WaveMetrics, Portland, OR, United States) or Excel. Violin plots were generated using the webtool from http://shiny.chemgrid.org/boxplotr.

## Results

### CAPS-1 Is Present at Synaptic and Extra-Synaptic Sites in Sensory Neurons

**FIGURE 1:**
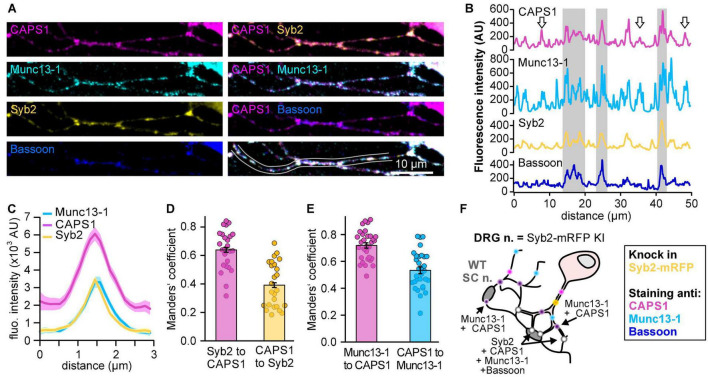
CAPS1 clusters are distributed over synaptic and extra-synaptic sites. **(A)** Representative neurite of a DRG neuron (DIV6) derived from synaptobrevin2-mRFP (yellow) knock-in mouse immuno-labeled with anti-CAPS1 (magenta), anti-active zone protein Munc13-1 (cyan) and anti-bassoon (blue) antibodies. **(B)** Line profiles of the fluorescence intensities along the neurite outlined in **(A)** in all four channels. The gray squares correspond to synapses identified by high anti-bassoon fluorescence. Arrows indicate extrasynaptic sites in which CAPS1 and Munc13- co-localize. **(C)** Average line profile diagram for CAPS1 over synapses selected according to co-localized synaptobrevin 2-mRFP, Munc13-1 and bassoon (not shown) signals (*n* = 81 synapses; *N*_*experiment*_ = 3). **(D)** Manders’ overlap coefficients of synaptobrevin 2-mRFP and CAPS1 represented as bar graph of the average ± SEM over the scatter dot plot of individual neurite values (*n* = 25 neurites; *N*_*experiment*_ = 3). **(E)** Manders’ overlap coefficients of Munc13-1 and CAPS1 represented as bar graph of the average ± SEM over the scatter dot plot of individual neurite values (*n* = 31 neurites; *N*_*experiment*_ = 3). **(F)** Schematic representation of the experimental design. Light blue dots indicate Munc13-1-positive puncta and yellow dots correspond to synaptobrevin 2-mRFP on SVs or LDCVs at extra-synaptic locations in DRG neurons. Cytosolic distribution of CAPS1 is shown in magenta while enrichment at heterotypic synapses is represented with white dots.

We hypothesize that the availability of CAPS1 at synapses could influence its role in priming SVs. Therefore, we first investigated synaptic and extra-synaptic localization of CAPS1 in DRG neurons co-cultured with their natural target cells the SC neurons by comparing its localization to Munc13-1, another well-established priming protein for SV and LDCV exocytosis in neurons (Varoqueaux et al., 2002; van de Bospoort et al., 2012). To identify heterotypic synaptic contacts formed between both type of neurons, we isolated DRG neurons from synaptobrevin 2-mRFP knock-in mice (Matti et al., 2013), co-cultured them with SC WT cells (Figure 1F), and stained them with antibodies against CAPS1, Munc13-1, and bassoon to identify the synapses (Farina et al., 2015; Shaib et al., 2018). CAPS1 showed a partially diffuse punctate distribution along the neurites (Figure 1A). A majority of these CAPS1 puncta co-localized with the synaptic marker bassoon as well as synaptic vesicle marker synaptobrevin 2 and the priming protein Munc13-1 (Figure 1C), indicating that they were localized to the heterotypic DRG/SC neuron synapses. Additionally, CAPS1 puncta were found at extra-synaptic sites, where they partially co-localized with Munc13-1 as indicated in the exemplary line profile (Figure 1B). The co-localization analysis performed on DRG neurites supported this finding. Only 39% of CAPS1 co-localized with synaptobrevin 2, but 53% with Munc13-1 (Manders’ coefficient of CAPS1 to synaptobrevin 2: 0.39 ± 0.03, *n* = 25, *N* = 3, and CAPS1 to Munc13-1: 0.53 ± 0.02, *n* = 31, *N* = 3; Figures 1D,E). Overall, these data show that endogenous CAPS1 is enriched at synapses but also in extra-synaptic clusters where it partially co-localizes with Munc13-1.

### Design and Functional Analysis of the CAPS1/2 Chimera Protein

**FIGURE 2:**
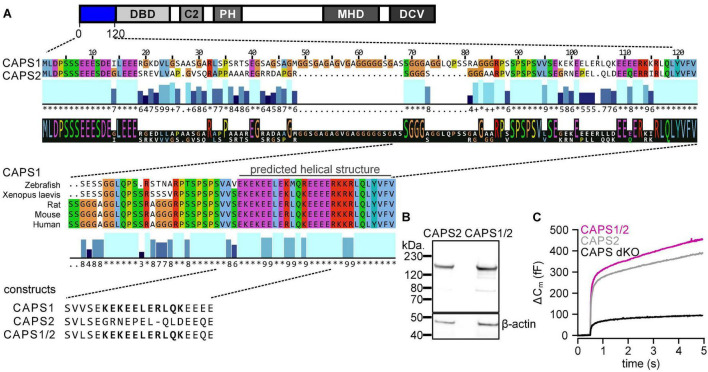
Identification of a distinct predicted coiled-coil structure in the N-terminus of CAPS1. **(A)** CAPS domain structure with N-terminal sequence alignment of CAPS1 and CAPS2 showing a unique CAPS1 sequence (upper panel), which is aligned with the same sequence in other species (lower panel). Conservation and consensus sequence are automatically calculated by jalview and displayed below the sequence alignment. Conservation is visualized as blue bars. Their tone and height indicate the score for each column. The numbers reflect the conservation of physico-chemical properties in the alignment: identities score highest, and the next most conserved group include substitutions of an amino acid to an other lying in the same physico-chemical class. Conserved columns are indicated by an * (score of 11 with default amino acid property grouping), and columns with mutations where all properties are conserved are marked with a + (score of 10, indicating all properties are conserved). The consensus sequence logo indicates with its size the relative number of amino acids per column. **(B)** Western blot analysis of CAPS2 and CAPS1/2 chimera protein expression. Chromaffin cells transfected with CAPS2b-HA or CAPS1/2-HA were lysed and blotted with an anti-HA antibody. β-actin was used as a loading control. **(C)** Averaged membrane capacitance measurements of CAPS dKO mouse chromaffin cells transfected with CAPS2b (light gray, *n* = 20) or CAPS1/2 (magenta, *n* = 22) compared to non-transfected controls (black, *n* = 15); *N*_*experiment*_ = 3.

Assuming that paralog-specific functions are mediated by protein-protein interactions, we hypothesize that these interactions are encoded in the most divergent regions of the protein. The sequence alignment of both murine paralogs (SwissProt: Q80TJ1 for CAPS1 and Q8BYR5 for CAPS2; Figure 2A) revealed that the N-terminal region (amino acids 1–120) contains the highest degree of variability between CAPS1 and CAPS2. In this region, highly conserved amino acid sequences are interrupted by three more divergent sequences encoded by amino acids 19–68, 74–81, and 98–108 in CAPS1. We further hypothesized that the sequences mediating protein function should be conserved between species. Hence, we compared the conservation of the aforementioned regions between CAPS1 homologs in other species. Only the amino acid region from 70 to 120 showed a high level of conservation. Finally, the biological functions of proteins are usually mediated by domains with a secondary structure. Therefore, we investigated the secondary structure of amino acid sequences 74–81 and 98–108 using the prediction tools provided by the SMART^1^, Pfam^2^, and AphaFold^3^ databases. Only the highly conserved unique CAPS1 region from amino acids 98–108 is located at the beginning of a predicted helical structure (over 80% confidence score). In contrast most of the corresponding sequence in CAPS2 does not form a helical structure. Interestingly, this 11 amino acids long CAPS1 sequence (98–108) contains a short sequence of 6 amino acids (EKEELE) that displays a high degree of similarity to a sequence of mSec7/Cytohesin, which has been shown to interact with Munc13-1 (Neeb et al., 1999). As CAPS1 and Munc13-1 are largely co-located in neurites (Figure 1), this similarity supported our approach.

To study the importance of this unique CAPS1 N-terminal sequence, we designed a CAPS1/2 chimera, in which we replaced amino acids 70–79 in CAPS2 with the 11 amino acids 98–108 of the CAPS1 N-terminus (CAPS1/2; Figure 2A). We confirmed the correct size of the CAPS1/2 chimera by comparing it with the CAPS2 WT protein by western blotting (Figure 2B). Furthermore, we verified its functionality by performing exocytosis rescue experiments in CAPS dKO mouse adrenal chromaffin cells using membrane capacitance measurements. We found that the CAPS1/2 chimera promoted exocytosis to the same extent as CAPS2b WT when overexpressed in dKO cells (Figure 2C). Therefore, the CAPS1/2 chimera was correctly expressed and fully functional.

### Characterization of Calcium-Dependent Activator Protein for Secretion-Halo Overexpression Level and Protein Distribution

**FIGURE 3:**
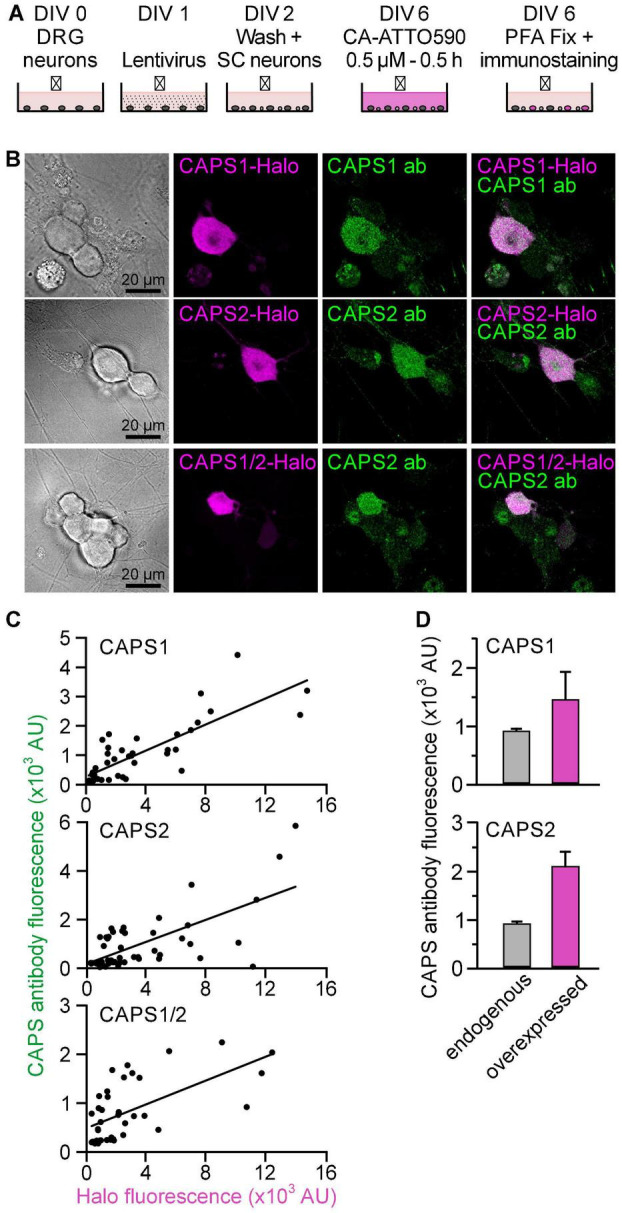
All CAPS-Halo constructs are equally expressed to twice the endogenous level. **(A)** Schematic staining protocol. DRG neurons were isolated from CAPS dKO mice and SC neurons were prepared from WT mice. **(B)** DRG CAPS dKO neurons expressing CAPS1-, CAPS2b-, or CAPS1/2 chimera-Halo stained with CA-ATTO590 (magenta) and immuno-labeled with anti-CAPS1 or CAPS2 antibody (green). **(C)** Correlation plot of CAPS1 or CAPS2 antibody fluorescence intensities against CA-ATTO 590 (*n* = 42, *n* = 52, and *n* = 39 for CAPS1, CAPS2, and CAPS1/2 chimera, respectively, *N* = 3). **(D)** Mean endogenous CAPS expression level measured in wild type neurons (gray bar) compared to overexpressed CAPS-Halo level (magenta bar). Top and bottom graph represent CAPS1 and CAPS2, respectively (*n* = 15 and *n* = 8 for endogenous vs. overexpressed CAPS1 and *n* = 16 and *n* = 18 for endogenous vs. overexpressed CAPS2, respectively; *N*_*experiment*_ = 1).

To analyze the role of the CAPS1 N-terminal sequence in protein localization, we used the HaloTag technology (Los et al., 2008) to visualize CAPS1, CAPS2b, and the CAPS1/2 chimera upon transfection via Lentivirus in dKO DRG neurons. We first evaluated the overexpression level of our three CAPS-Halo constructs by staining the neurons on DIV6 with CA-ATTO590 (magenta) and by immunolabeling using anti-CAPS1 or anti-CAPS2 antibody (green) immediately thereafter (Figures 3A,B). CA-ATTO590 staining was specific as non-transfected neurons that lacked the anti-CAPS signal but were visible in the bright-field images were devoid of CA-ATTO590 (Figure 3B). More importantly, the correlation plot of the fluorescence intensity of the labeling with the anti-CAPS1 or anti-CAPS2 antibodies and CA-ATTO590 showed a linear relationship between both stains (Figure 3C). This indicates that, using our staining protocol, the CA-ATTO590 fluorescence signal intensity is a good indicator for the CAPS-Halo construct expression level. In addition, the comparative analysis of endogenous versus overexpressed protein levels showed that 5 days after viral transduction the amount of CAPS-Halo constructs were, on average, not more than twice as high as that of the endogenous protein (Figure 3D).

**FIGURE 4:**
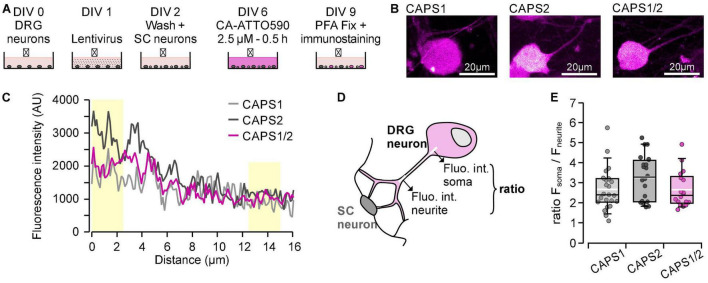
CAPS2 accumulates in the cell soma, while CAPS1 and CAPS1/2 chimera move into the neurites. **(A)** Schematic staining protocol. DRG and SC neurons were isolated from CAPS dKO and WT mice, respectively. **(B)** Representative DRG neuron somata transfected with either CAPS-Halo construct and stained with CA-ATTO590. **(C)** Line scan analysis performed on the neurons shown in **(B)**. The lines were drawn from 3 μm in the soma to 13 μm in the neurites and had a width of three pixels. Yellow boxes correspond to the portion of the line used for the analysis depicted in **(D)**. **(D)** Schematic representation of the method used to determine the distribution of CAPS-Halo from the soma in the neurite. A fluorescence intensity profile of Halo ligand CA-ATTO590 (magenta) was measured along a 15 μm long line shown in white. The relationship between somatic and neurite fluorescence was calculated as a ratio between the fluorescence intensities over the first and last 2.5 μm on the line (thick ends). **(E)** Ratios of CA-ATTO590 soma vs. neurite fluorescence intensity in neurons expressing CAPS1-, CAPS2b-, or CAPS1/2 chimera-Halo. Box plots display median (black line) and mean (white line). *n* = 23, *n* = 20, and *n* = 18 for CAPS1, CAPS2b, and CAPS1/2, respectively, *N*_*experiment*_ = 4. The differences between CAPS2b and CAPS1 or CAPS1/2 chimera are not significant.

Then we investigated the CAPS-Halo transport from the cell soma to distant synapses in the CAPS dKO DRG neurons. We designed the staining protocol with CA-ATTO590 with a delay of 3 days between cells staining and visualization. This delay ensured that the stained CAPS-Halo proteins had sufficient time to reach their target, while the newly formed unstained proteins did not interfere with the measurements during their transport along the neurites (Figure 4A). All three proteins were detected diffusely in the soma and the neurite, but CAPS2b was consistently more abundant in the cell soma than in the neurite (Figures 4B,C). Figure 4D schematically illustrates the distribution of CAPS (magenta) in the soma and neurites and the analysis method. The mean intensity of CAPS2b in the soma was three times higher than that in the neurite (ratio = 3.28 ± 0.25, *n* = 20, Figure 4E). The CAPS1 soma versus neurite fluorescence ratio was comparatively lower (2.67 ± 0.22, *n* = 23). Interestingly, the CAPS1/2 chimera showed a phenotype identical to that of CAPS1 with a mean fluorescence intensity ratio of 2.65 ± 0.21 (*n* = 18, Figure 4E), indicating a potential role for the CAPS1 N-terminus in the distribution of the protein.

### A Unique CAPS1 N-Terminal Sequence Determines CAPS1 Localization at Synapses

**FIGURE 5:**
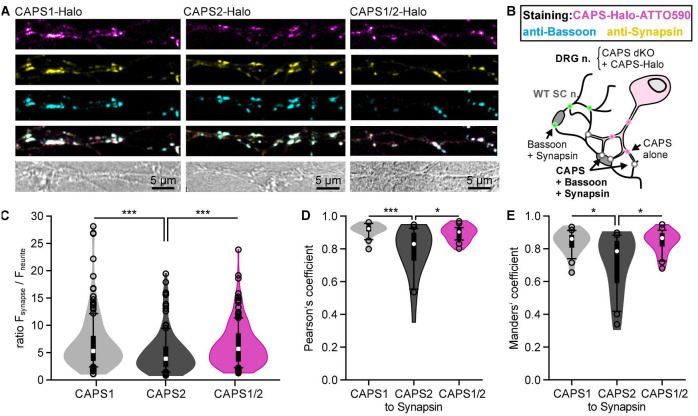
The N-terminal sequence of CAPS1 determines its pre-synaptic localization. **(A)** Representative neurites of DRG CAPS dKO neurons transfected with CAPS1-, CAPS2b-, or CAPS1/2 chimera-Halo. On DIV2 SC neurons were added to the cultures. Halo ligand CA-ATTO590 labeling (magenta) on DIV6. On DIV9 cells were fixed and immuno-labeled with specific antibodies against bassoon (cyan) and synapsin (yellow). Note that the lookup table in the upper row (CAPS-Halo) is magenta hot, with white representing the highest fluorescence. This lookup table was not used in the merged image in the third row to correctly represent co-localization. All images for individual channels are scaled to the same values. **(B)** Schematic representation of the experimental design. Green dots indicate homotypic synapses from SC neurons marked by a positive staining of synapsin and bassoon, while co-localization of CAPS-Halo constructs with synapsin and bassoon are visualized as white dots. **(C)** Violin plot of CAPS-Halo normalized fluorescence intensity ratio for all three constructs. Synaptic CA-ATTO590 fluorescence intensity was normalized to the signal in adjacent areas of the neurites. *n* = 182, *n* = 148, and *n* = 173 synapses for CAPS1, CAPS2b, and CAPS2/1 chimera, respectively; *N*_*experiment*_ = 3; ***p < 0.001 one-way ANOVA on ranks with Dunn’s *post hoc* test. **(D)** Co-localization of CAPS1, CAPS2b, and CAPS1/2 with synapsin was measured on isolated synapses of DRG neurites and quantified using the Pearson’s correlation coefficient. *n*_*neurites*_ = 24; *N* = 3 for all constructs; *p = 0.010 or ***p < 0.001 one-way ANOVA on ranks with Tukey *post hoc* test. **(E)** Manders’ overlap coefficients for the proportion of CAPS1, CAPS2b, and CAPS1/2 immunoreactivity in synapsin-positive locations. *n*_*neurites*_ = 24; *N*_*experiment*_ = 3 for all constructs; *p = 0.014 for CAPS1 and CAPS2 or *p = 0.010 for CAPS1/2 and CAPS2 one-way ANOVA on ranks with Tukey *post hoc* test. Box plots are superimposed on the violin plots. Median is shown as white dot while outliers are represented as empty circles.

We then directly compared the localization of overexpressed CAPS1, CAPS2b, and the CAPS1/2 chimera at synapses of DRG neurons from CAPS dKO mice. We used the same experimental protocol illustrated in Figure 4A, and the synapses were marked by co-immunostaining with anti-synapsin and anti-bassoon antibodies (Micheva et al., 2010; Figure 5B). Similar to the endogenous CAPS1 protein localization (Figure 1), overexpressed CAPS-1 displays a cytosolic diffuse staining superseded with punctate staining in the neurites. These puncta showed a high degree of co-localization with the presynaptic markers synapsin and bassoon (Figure 5A). In contrast, CAPS2b cytosolic staining was more pronounced and the punctate staining was less intense than that of CAPS1. Therefore, the over-expressed CAPS-Halo constructs recapitulate the localization of endogenous CAPS1 and CAPS2b in DRG neurons (Shaib et al., 2018). Interestingly, the CAPS1/2 chimera displayed a similar synaptic enrichment as CAPS1 (Figure 5A). To investigate the synaptic accumulation of CAPS1, CAPS2b, and CAPS1/2 chimera at bassoon- and synapsin-positive synapses, we measured CA-ATTO590 fluorescence intensity at the synapse and normalized it to its fluorescent intensity in the neurite. We found that overexpressed CAPS1/2 chimera recapitulated the synaptic accumulation of overexpressed CAPS1, which was significantly higher than that of overexpressed CAPS2b (mean normalized fluorescence intensity: 6.54 ± 0.34, 4.85 ± 0.30, and 6.29 ± 0.30 for CAPS1, CAPS2b, and CAPS1/2, respectively, Figure 5C). Co-localization analysis of the different CAPS constructs with the synaptic marker synapsin supported the results. Both CAPS-1 and CAPS1/2 chimera CA-ATTO590 labeling at synapsin-positive synapses, revealed a significantly higher degree of co-localization compared to CAPS2b (Manders’ overlap coefficients at synapses: 0.84 ± 0.01, 0.71 ± 0.04, and 0.84 ± 0.01 for CAPS1, CAPS2b, and CAPS1/2, respectively, vs. synapsin, *n* = 24, *N* = 3, Figure 5E). Hence, introducing the unique CAPS-1 N-terminal sequence into the CAPS2b protein alters its subcellular localization, leading to increased synaptic enrichment.

### CAPS1/2 Chimera Shows an Enhanced Localization to Active Zones

**FIGURE 6:**
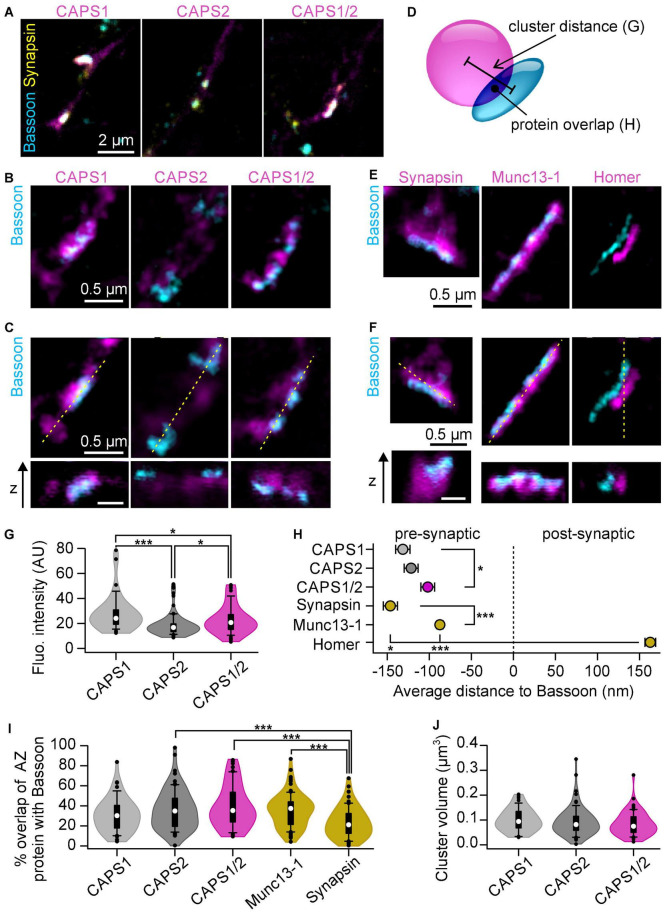
3D STED microscopy resolves CAPS1 N-terminus function in CAPS1 accumulation near active zones. **(A–C)** DRG CAPS dKO neurons infected with CAPS1- CAPS2b or CAPS1/2 chimera-HaloTag and co-cultured with WT SC neurons. Vital staining of the cells with HaloTag ligand CA-ATTO590 (magenta) was conducted on DIV6 followed by immunostaining for bassoon (cyan) and synapsin (yellow) on DIV9. **(A)** Representative confocal overviews of neurite networks for each CAPS construct. Enlarged portion of a synapse outlined in **(A)** acquired with 2D **(B)** or 3D **(C)** STED microscopy. The 3D STED images display a sagittal and an axial section of the synapse. The orientation of the axial section is indicated as a dashed yellow line on the sagittal section. Scale bar is 0.5 μm for all STED images. **(D)** Schematic representation of image analysis. 3D protein clusters were defined on the 3D STED images. Their center of mass was used to calculate the distance between CAPS and bassoon clusters. Further, their degree of overlap and volume was quantified. **(E,F)** DRG/SC WT neuron co-culture immunostained for bassoon and either synapsin, Munc13-1, or Homer. Representative 2D **(E)** and 3D **(F)** STED micrographs. Scale bar is 0.5 μm. **(G)** Average fluorescence intensities within the defined CAPS clusters. **(H)** Mean distance between the center of mass of the indicated protein clusters relative to bassoon cluster (*p = 0.038 for CAPS1 CAPS2b and CAPS1/2, *p = 0.048 for synapsin and Homer or ***p < 0.001). **(I)** Percentage of indicated protein cluster overlapping with bassoon (***p < 0.001). **(J)** Analysis of CAPS cluster volume. CAPS1 *n* = 48 CAPS2b, *n* = 78, and CAPS1/2 *n* = 59 synapses (*N*_*experiment*_ = 3); Munc13-1 *n* = 83; synapsin *n* = 61 and Homer *n* = 64 synapses (*N* = 1). Statistical significance determined by one-way ANOVA on ranks followed by Dunn’s *post hoc* test. The violin plots include a box plot with the median shown as a white dot, and outliers as black dots.

To go beyond the finding that the CAPS1 N-terminus induces its synaptic accumulation, we were interested in whether it had an impact on the sub-synaptic localization of the protein. We performed STED super-resolution microscopy and compared the localization of CAPS-Halo constructs to the active zone marker bassoon (Lee et al., 2003; Nishimune et al., 2016). DRG neurons from CAPS dKO mice transfected with CAPS1- CAPS2b- or CAPS1/2 chimera-Halo were stained with CA-ATTO590 and immunolabeled as shown in Figures 4A, 5B, respectively. After identifying synapses by co-localization of CAPS, bassoon, and synapsin in the confocal overview (Figure 6A), we acquired two color (CAPS and bassoon) 2D STED images and 3D STED image stacks of a smaller region around the identified synapses (Figures 6B,C). At these synapses, bassoon appeared as a narrow band, marking exclusively the active zone, whereas the CAPS signal was distributed in a much larger portion of the pre-synapse. To contextualize our data, we compared the synaptic distribution of CAPS-Halo with the synaptic vesicle marker synapsin, the active zone protein Munc13-1, and the postsynaptic marker Homer1 (Figures 6E,F). Their sub-synaptic localization was very similar to that reported for hippocampal neurons (Figures 6H,I; Grauel et al., 2016). Hence, our 3DSTED acquisition was particularly suited to resolve fine differences in the localization of synaptic proteins.

The fluorescence intensity of CAPS cluster was highly dependent on the over-expressed construct. While CAPS1 clusters were 1.5 times brighter than CAPS2 clusters, CAPS1/2 chimera clusters displayed an intermediate fluorescence intensity (Figure 6G). These findings corroborate our confocal microscopy data showing that CAPS1 N-terminus plays an important role in accumulating the protein in synapses. The in-depth localization analysis of all three CAPS constructs revealed that the distance between the center of mass of CAPS and bassoon clusters was significantly smaller for the CAPS1/2 chimera than for CAPS1 (Figure 6H). However, contrary to our expectations CAPS2b had an intermediate position between CAPS1 and CAP1/2. Additionally, the measurement of CAPS, synapsin, or Munc13-1 clusters overlap with bassoon clusters, indicate that CAPS1 behaves like the SV-associated protein synapsin, while CAPS2b and the CAPS1/2 chimera resembles the active zone protein Munc13-1 (Figure 6I). These different localizations of the CAPS1, CAPS2b, and CAPS1/2 chimera are not due to differences in cluster size (Figure 6J). Taken together, our data show that CAPS1 clusters were intermediately positioned in the synapse between synapsin and Munc13-1 and that the CAPS1/2 chimera was significantly closer to the active zone than CAPS1. Therefore, the CAPS1 unique N-terminal sequence is involved in CAPS1 enrichment at pre-synapses but our data suggests that an unknown sequence of CAPS2 might over-proportionally increases the localization of CAPS1/2 chimera toward the active zone.

### CAPS2 Dynamics at Synapses Is Reduced in Comparison to CAPS1 and the CAPS1/2 Chimera

**FIGURE 7:**
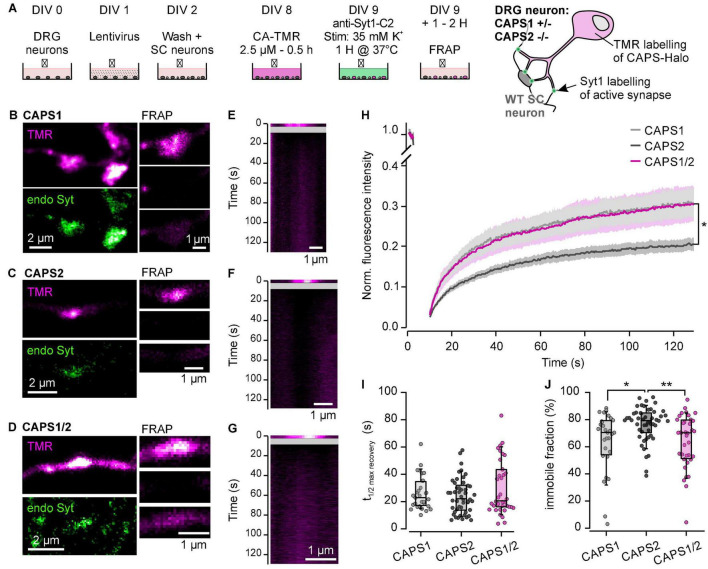
N-terminal sequence of CAPS1 induces its specific transport in synapses. **(A)** Schematic representation of the staining protocol (left). DRG neurons were isolated from CAPS1^+/–^ CAPS2^–/–^ mice and co-cultured with WT SC neurons and transfected with lentiviruses encoding for the respective CAPS construct on DIV1. Vital staining of the cells with bleachable HaloTag ligand CA-TMR (magenta) was conducted on DIV8. On DIV9 the neurons were incubated for 1 h in weak depolarizing conditions (35 mM K^+^) with an antibody directed against the luminal domain of synaptotagmin1 coupled to Sulfo-Cyanine 2 (Syt1-C2). This antibody is taken up by endocytosis in active synapses that are then labeled in green. The neurons were allowed to recover for 1 h at 37°C before proceeding with the FRAP experiment. The result of the staining protocol is schematically shown on the right. **(B–D)** Left are shown the labeling of the synapse with HaloTag ligand CA-TMR (magenta) and with Syt1-C2 (green). On the right are shown 3 snapshots of the selected synapse just prior to photo-bleaching (top), just after (center) and 120 s (bottom) after photo-bleaching. Representative synapses for neurons transfected with CAPS1-, CAPS2b- and CAPS1/2-Halo are displayed in **(B–D)**, respectively. **(E–G)** Kymogram representation of the FRAP recording shown in **(B–D)**, respectively. The line scan was made through the synapse parallel to the neurite. **(H)** Average fluorescence recovery over time of synapses in DRG neurons expressing CAPS1- (light gray), CAPS2b- (dark gray) and CAPS1/2-Halo (magenta). *p = 0.047 and 0.022 for CAPS1 and CAPS1/2 vs. CAPS2, respectively. **(I)** Half time recovery of maximal fluorescence represented as box plot super imposed with the values of individual cells. **(J)** Immobile CAPS fraction displayed as box plot super imposed with the values of individual cells. *p = 0.018 and **p = 0.005 for CAPS1 and CAPS1/2 vs. CAPS2, respectively. Number of measured synapses from either native CAPS dKO neurons or expressing either CAPS1, CAPS2b, or CAPS1/2 chimera was 28, 50, and 36 respectively, in three independent experiments.

The specific accumulation of CAPS1 and CAPS1/2 at synapses could indicate a different dynamic behavior of these proteins compared to CAPS2. We examined this hypothesis with FRAP experiments. The CAPS-Halo constructs were labeled with the bleachable fluorophore TMR (Morisaki and McNally, 2014) 1 day prior to the experiment to ensure a large mobile pool of CAPS. In order to identify active synapses in living cells, we used an anti-body directed against the luminal domain of synaptotagmin1 coupled to Sulfo-Cyanine 2, which we applied to the neurons under depolarizing conditions (Truckenbrodt et al., 2018; Figure 7A). The synapses showing a signal for both proteins were selected for FRAP experiments (Figures 7B–D). The recovery phase after photobleaching was relatively long for all three CAPS proteins as a plateau phase was reached only at about 120 s (Figures 7E–H). In comparison, the monomeric red fluorescent protein (mRFP) reached it in about 50 s (Supplementary Figure S1). The half-time of recovery of all CAPS proteins was close to 25 s (Figure 7I). Similarly, the mobile fraction of all three CAPS proteins was much smaller than that of mRFP indicating that none of the CAPS proteins are freely diffusible (Figures 7H,J compared to Supplementary Figures S1A,B). Remarkably, the mobile fraction of CAPS2b was significantly smaller than that of CAPS1 and CAPS1/2. After 120 s, CAPS2b recovered only 22.3 ± 1.6% of its pre-flash fluorescence intensity, while CAPS2 and CAPS1/2 recovered by 33.6 ± 3.8 and 32.6 ± 3.2%, respectively (*n* = 28, 50, 36 for CAPS1, CAPS2b and CAPS1/2 respectively; *N* = 3; Figure 7H). Consequently, the immobile fraction of CAPS2b was 1.2 time higher than that of CAPS1 of CAPS1/2b (Figure 7J). This data indicates that CAPS1 is not retained at synapses more than CAPS2. Rather, its transport into synapses is increased in comparison to CAPS2. Furthermore, the unique N-terminal sequence of CAPS1 increased the mobility of CAPS2 exactly to the level of CAPS1, indicating that this sequence might be involved in the interaction with transport proteins.

### Synaptic Vesicles Exocytosis Is Partially Rescued by the CAPS1/2 Chimera

**FIGURE 8:**
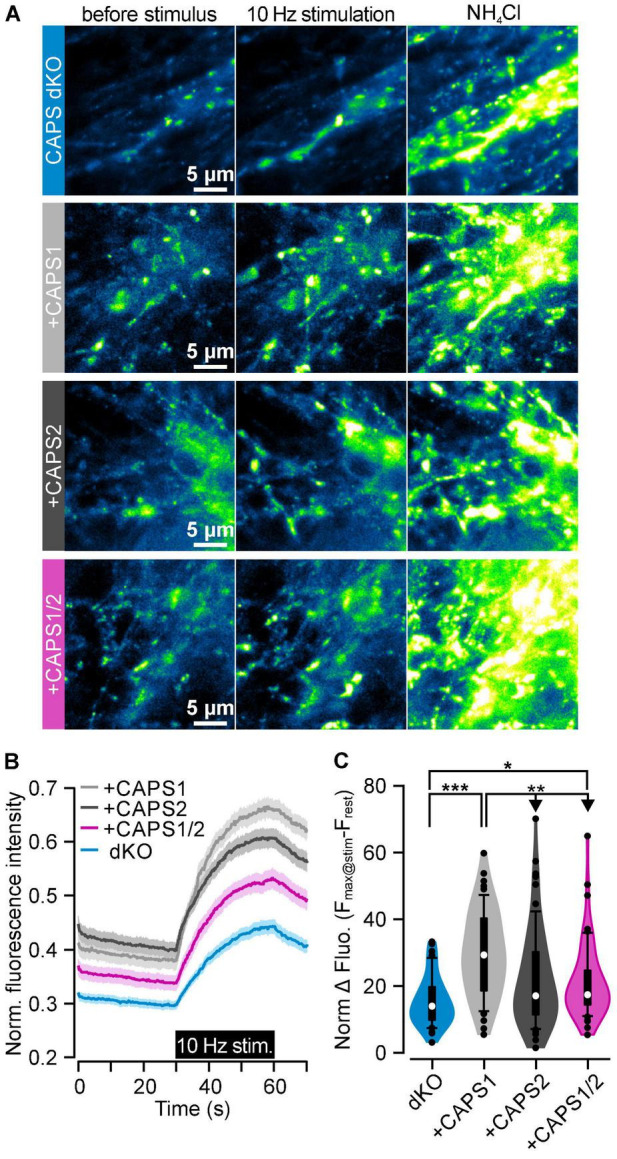
CAPS1/2 chimera partially rescues SV exocytosis. **(A)** CAPS dKO DRG neurons were isolated from E18/19 mice, infected with lentiviruses encoding for SypHy and either CAPS1-Halo, CAPS2b-Halo, or CAPS1/2 chimera-Halo and co-cultured with WT SC neurons for 8 days. Displayed from left to right are representative images of SypHy signal in DRG neurons before and during 10 Hz field electrode stimulation and upon NH_4_Cl application for full de-quenching of SypHy vesicular pool. From top to bottom are representative CAPS dKO neurons that were or were not co-transfected with either CAPS1, CAPS2b, or the CAPS1/2 chimera construct. **(B)** Normalized SypHy signal at synapses in response to electrical stimulation for CAPS dKO neurons (blue), expressing CAPS1 (light gray), CAPS2b (dark gray), or CAPS1/2 chimera (magenta). SypHy fluorescence intensity of each synapse was normalized to its fluorescence intensity during NH_4_Cl application. Data are mean ± SEM. **(C)** Violin plot of the maximum normalized SypHy fluorescence intensity increase at each synapse elicited by 10 Hz electrical stimulation. Experiments were performed on a minimum of three independent cultures for every genotype. Number of measured synapses from either native CAPS dKO neurons or expressing either CAPS1, CAPS2b, or CAPS1/2 chimera was 45, 53, 64, and 58, respectively, in more than 3 independent experiments. *p < 0.05, **p < 0.01, or ***p < 0.001 ANOVA on rank with Dunn’s *post hoc* test.

Since the CAPS1 and CAPS1/2 chimera localized similarly well to synapses compared to the CAPS2b, we expected that the CAPS1/2 chimera would have a similar function in synaptic transmission as CAPS1. To test this hypothesis, we measured synaptic transmission between DRG neurons and SC neurons using the SypHy-based imaging method (Miesenbock et al., 1998; Granseth et al., 2006). Active synapses responded to the field electrode stimulation by an increase in SypHy brightness (Figure 8A, middle). Data were normalized to the total SV pool visualized upon NH_4_Cl treatment (Figure 8A, right). We first verified that the deletion of CAPS1, not CAPS2, reduced synaptic transmission in DRG neurons (Supplementary Figure S2; Shaib et al., 2018), and then measured the synaptic transmission in CAPS dKO neurons transfected with the CAPS constructs. The overexpression of CAPS1 fully rescued synaptic transmission in CAPS dKO neurons (Figures 8B,C). CAPS2b and CAPS1/2 chimera overexpression resulted in a stronger SypHy fluorescence increase during stimulus as compared to CAPS dKO neurons. However, this increase was significant only in CAPS1/2 chimera overexpressing neurons (*p* = 0.034, n_*synapse*_ was 45 and 58 for CAPS dKO and CAPS1/2 overexpressing neurons, respectively). Hence, only the CAPS1/2 chimera, but not CAPS2b, rescued synaptic transmission in CAPS dKO neurons, but to a much smaller extent as CAPS1. These findings suggest that the correct localization of the protein at the synapse is important for CAPS function, but that the specificity of CAPS1 SV priming function is probably encoded by a different domain of the protein.

### Identification of Putative CAPS1-Interacting Proteins

**FIGURE 9:**
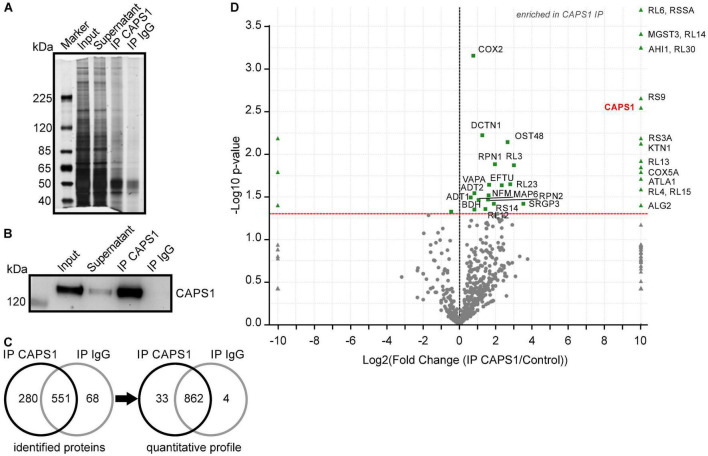
Immunoprecipitation and mass spectrometry analysis of CAPS1. **(A)** Representative silver-stained proteins on a SDS gel from input, supernatant, CAPS1 IP, and IgG control IP. **(B)** Representative western blots of CAPS1 with the indicated bands for input, supernatant, CAPS1 IP, and IgG control IP. **(C)** Total spectrum count analysis of proteins identified in three independent eluates after CAPS1 IPs and IgG control IPs by nano-LC-MS/MS (minimum of 2 peptides/protein). Left: number of protein identifications, right: semi-quantitative profile showing proteins significantly enriched (*n* = 3 for CAPS1 and IgG, *t*-test, *p* < 0.05). **(D)** Volcano plot of *p*-value vs. x-fold change of identified peptide spectrum counts. Proteins that were significantly enriched with CAPS1 or IgG antibodies with a *p*-value < 0.05 (*t*-test) are highlighted in green. The CAPS1 protein (red) is exclusively identified in all CAPS1 IPs from whole mouse brain lysates (*n* = 3, biological replicates for CAPS1 IPs and IgG IPs).

To identify which proteins could be involved in CAPS1 preferential synaptic localization, we performed CAPS1 immunoprecipitations (IPs) analyzed by western blot and MS. Silver-staining verified that the total protein amount for CAPS1 IP was higher as compared with its respective IgG control IPs (Figure 9A), while western blotting confirmed the accumulation of CAPS1 after IP (Figure 9B). The band of CAPS1 protein was detected at about 145 kDa, and the specific accumulation was validated by the absence of the signal in the IgG control IP. In addition, MS analysis of the CAPS1 supported specificity by reliably identifying the most abundant protein hits with a sequence coverage of 54%, whereas it was completely absent in the IgG control IP (Figure 9D and Supplementary Figure S3). In addition to CAPS1, 898 proteins were enriched in the CAPS1 IP. To further extract putative CAPS1-interacting proteins with high confidence, a semi-quantitative profile was constructed showing proteins with significant enrichment (Figures 9C,D and Supplementary Figure S3). This analysis revealed 32 likely CAPS1-interacting partners and CAPS1 itself. Proteins involved in intracellular transport processes such as MAP6, kinectin, and jouberin were identified and may play a role in the localization of CAPS1 (Figure 9D).

Interestingly, the MS analysis of CAPS1 IP identified two CAPS interaction partners already described in the literature, supporting this approach for the discovery of new interaction partners for CAPS1. Among them, the transport protein dynactin subunit 1 was significantly enriched. Dynactin subunit 1 interacts with CAPS2 and affects axonal CAPS protein distribution (Sadakata et al., 2007a). We now suggest that it interacts with CAPS1 as well. In addition, we identified DmX-like protein 2, also called rabconnectin-3, which was not significantly enriched but was 3.6 fold higher in the CAPS1 IP compared to the IgG control IP. Rabconnectin-3 has been shown to interact with CAPS1, enabling the recruitment of soluble CAPS1 to DCV membranes to function in vesicle acidification (Crummy et al., 2019). Its contribution to CAPS1 localization at pre-synapses is probable but remains to be demonstrated. The previously reported and newly identified putative CAPS1-interacting proteins need to be further analyzed to shed light on the molecular function of CAPS1.

## Discussion

Herein, we investigated the subcellular localization of CAPS paralogs in mammalian sensory neurons by overexpressing CAPS-Halo via the lentiviral transfection system in CAPS dKO neurons, which has several advantages: Working in a clean genetic background reduced potential interferences from endogenous CAPS protein, which was particularly important because not all DRG neurons express both CAPS paralogs. Indeed, only peptidergic DRG neurons express CAPS1 and CAPS2, while non-peptidergic neurons express only CAPS1 (Shaib et al., 2018). We used the HaloTag technology (Los et al., 2008) instead of classical fluorescent protein to visualize the CAPS constructs since it allowed for a high degree of freedom in designing the experimental protocol. The HaloTag is intrinsically non-fluorescent and is made visible in living cells only by staining with a membrane-permeable dye. These fluorescent dyes exist in a variety of colors and are more or less photostable; hence, we could adapt our staining protocol easily to perform confocal, STED microscopy or FRAP experiments. Furthermore, we visualized either the entire pool of CAPS-Halo or only the proteins at their final position by using a pulse type staining protocol. Finally, the lentiviral system is best suited for the long-term expression of the desired proteins in neurons with low cytotoxicity (Naldini et al., 1996; Hioki et al., 2007; Lundstrom, 2019).

CAPS1 and CAPS2 share approximately 80% amino acid sequence identity and contain the same functional domains, but are functionally distinct. While CAPS1 alone could promote SV priming (Supplementary Figure S2; Shaib et al., 2018) CAPS2 promotes LDCV exocytosis in neurons (Sadakata et al., 2004; Jockusch et al., 2007; Farina et al., 2015; Shaib et al., 2018). Consistent with this functional difference, we demonstrated that CAPS1 is localized preferentially at pre-synapses while CAPS2 is expressed more uniformly throughout the cell. We have also identified a short, highly conserved sequence of 11 amino acids at the N-terminus of CAPS1, which when inserted in CAPS2b, can direct the CAPS1/2 chimeric protein to pre-synapses. Furthermore, the mobility of the CAPS1/2 chimera in synapses matched exactly the mobility of CAPS1. Our data suggests that this unique sequence might be involved in the transport of CAPS1 into the pre-synapse. Intriguingly, the CAPS1/2 chimera was significantly closer to bassoon than CAPS1 while CAPS2 occupied an intermediate position. Thus although the N-terminal sequence of CAPS1 induces the accumulation of CAPS1 at synapses a specific unknown sequence of CAPS2 appeared to better localize the chimera to the active zone. Furthermore, the overexpression of CAPS1/2 chimera in CAPS dKO neurons induced some recovery of synaptic transmission but not to the level reached upon CAPS1-Halo overexpression, indicating that the functional differences between both proteins are not solely attributed to their different subcellular localizations. Most likely, further sequence and structural divergences between both CAPS paralogs are involved in the specific role of CAPS1 in priming SVs. The remarkable role of the N-terminal sequence in the accumulation of CAPS1 at the pre-synapses is reminiscent of Munc13s synaptic localization, which is mediated by interactions of their N-termini with the active zone proteins. Indeed, the ELKS1-dependent targeting of bMunc13-2 to synapses is facilitated by its N-terminal predicted coiled-coil region (Kawabe et al., 2017). Likewise, ubMunc13-2 and Munc13-1 are recruited to pre-synapses by similar interaction mechanisms with RIM (Betz et al., 2001; Dulubova et al., 2005; Andrews-Zwilling et al., 2006).

The differences between the synaptic accumulation of CAPS1 or CAPS1/2 chimera and CAPS2b were significant but not as large as expected. The normalized fluorescence of CA-ATTO590 at synapses was approximately 1.30 times higher in CAPS1- or CAPS1/2 chimera-Halo than in CAPS2b-Halo expressing cells. One possibility is that another region of the protein is also involved in its transport to the synapse. Synaptic localization of CAPS1 in hippocampal neurons are associated with the C-terminal region containing the DCV and MHD domains (van Keimpema et al., 2017). Recently a C-terminal amino acid at position 1252 that is subject to RNA editing from glutamate-to-glycine has been shown to be involved in CAPS1 synaptic localization (Shumate et al., 2021). However, the C-terminal region is highly conserved and has a similar domain structure in both CAPS paralogs (Speidel et al., 2003). Moreover, the sequence containing the glutamate subject to RNA editing is found in both proteins. Thus, this region could induce a re-localization from the soma to the neurites of both isoforms, but it is unlikely that this C-terminal region is responsible for the specific pre-synaptic enrichment of CAPS1. During visual inspection of the stained samples, identifying neurites stained with CA-ATTO590 in CAPS2b-Halo transfected cells was more challenging than cells transfected with the other two constructs. This difference was not due to different transfection efficiencies since a similar amount of stained soma was observed for all constructs (Figure 3). Instead, the fluorescence intensity of neurites (Figure 4C) in cells expressing CAPS2b-Halo was lower than that in CAPS1 or CAPS1/2 chimera, as indicated by our measurements. Consequently, the fluorescent intensity of neurites in cells transfected with CAPS2b-Halo was at the limit of detection, which artificially skewed our results to higher values. In CAPS1-Halo or CAPS1/2 chimera-Halo transfected cells the neurites were easily visible, and the normalized fluorescent intensity values of individual synapses showed a broader distribution. Hence, we probably underestimated the difference in synaptic accumulation between CAPS1 or CAPS1/2 chimera and CAPS2b.

Using FRAP experiments, we showed that CAPS1 was clearly more mobile at synapse than CAPS2 indicating that CAPS1 was more actively transported to synapse than CAPS2. In contrast to all other experiments, the FRAP study was performed with DRG neurons from CAPS1^+/–^ CAPS2^–/–^ mice. The question that one might ask is whether the pool of endogenous non-labeled CAPS1 might specifically interfere with the mobility of the over-expressed CAPS1-Halo protein. As has been shown by Speidel et al. (2005) the amount of CAPS1 expressed in heterozygote cells is much lower than in CAPS1 WT cells. Thus the likelihood that the endogenous protein affects the fluorescent recovery of CAPS1-Halo is reduced. Moreover, the fluorescence recovery of the CAPS1/2-Halo reproduced exactly that of CAPS1-Halo. The chimera protein is to more than 99% identical to CAPS2. Therefore, if the endogenous CAPS1 protein affects the fluorescence recovery of our CAPS-Halo proteins then it should do it similarly to all of them. Consequently, the difference between CAPS1 and CAPS2 mobility at synapses it specifically due to the N-terminal sequence of CAPS1. This sequence did not affect the speed of recovery but rather it increased the pool of mobile CAPS1 by more than 50% in comparison to CAPS2. This might indicate that CAPS2 and not CAPS1 is retained in the synapse through specific biding to a presynaptic protein. This hypothesis is also supported by the fact that CAPS1/2 chimera is located closer to the active zone than CAPS1. However, CAPS1 is clearly more enriched at synapses than CAPS2 (Figures 5, 6). Thus, it appears that an increased transport of CAPS1 to the synapse is responsible for its synaptic accumulation.

CAPS1 IP in combination with MS analyses identified putative interaction partners that could be involved in pre-synaptic CAPS1 enrichment, including transport proteins like MAP6, kinectin, and jouberin, as well as the known CAPS interaction proteins dynactin subunit 1, which is also involved in transport, and rabconnectin-3. Interestingly, the dynactin subunit 1 and rabconnectin-3 binding regions were previously mapped to the N-terminus of CAPS (Sadakata et al., 2007a; Crummy et al., 2019). Dynactin subunit 1 is the most fully characterized subunit of the dynactin complex (Schroer, 2004) associated with axonal transport (Waterman-Storer et al., 1997). Additionally, dynactin is not only required for the retrograde transport activity of dynein but also for the anterograde transport activity of kinesin II (Deacon et al., 2003). In *Drosophila*, dynactin subunit 1 is required for coordination of bidirectional axonal transport at the synaptic termini, thereby affecting synaptic transmission (Lloyd et al., 2012). However, the dynactin binding domain can be found in both CAPS1 and CAPS2, and dynactin subunit 1 interacts with CAPS2 (Sadakata et al., 2007b). Thus, CAPS1 binding to dynactin may not induce a specific enrichment of CAPS1 at the pre-synaptic region. In contrast, specific binding of rabconnectin-3 to the N-terminal region of CAPS1 (amino acids 1–378) was recently reported (Crummy et al., 2019). Rabconnectin-3 consists of two subunits, α and β, which are both associated with synaptic vesicles. Rabconnectin-3β directly binds Rab3 GEP and thereby might recruit the protein from the cytosol to SVs where it has a function in the Ca^2+^-dependent exocytosis of neurotransmitters. After Rab3 GEP exerts its function, it may be released from the vesicles to the cytosol; thus, the binding of Rab3 GEP to rabconnectin-3 may be temporally regulated (Nagano et al., 2002; Kawabe et al., 2003). A similar transient interaction between CAPS1 and rabconnectin-3 to localize CAPS1 to SVs during priming is conceivable. This hypothesis is supported by the finding that in hippocampal neurons, CAPS1 is transiently displaced from the synapse into neurites upon intense stimulation as the pool of primed SVs is depleted (Farina et al., 2015). Further investigations are warranted to analyze the exact molecular mechanism of differential CAPS1 and CAPS2 transport and accumulation at synapses. In particular, the IP analysis of CAPS1 should be compared to the same analysis performed with CAPS2. Unfortunately, all attempts at generating valid CAPS2 IP failed possibly due to low affinity of CAPS2 anti-body in combination with low expression level of CAPS2 in comparison to CAPS1. Nevertheless, our findings about potential interaction partners involved in CAPS1 transport protein will open new avenue of investigations in CAPS function.

## Conclusion

Our data suggest an important role in the pre-synaptic localization of CAPS1 to the N-terminal part of the protein. While the conservation in the C-terminal priming domain(s) allows CAPS1 and CAPS2 to prime LDCVs with similar efficiency, the presence of the unique N-terminal localization signal in CAPS1 facilitates its function in SV priming. Interestingly, identical or similar sequences are also found in MAP7 and Cytohesin-1, which are involved in transport and neurotransmitter release. Hence, we suggest that this novel domain plays a more general role in protein localization.

## Data Availability Statement

The mass spectrometry proteomics data are available through the ProteomeXchange Consortium via the PRIDE partner repository with the dataset identifier PXD031625. Additional data and information that support the findings of this study are available from the corresponding author upon reasonable request.

## Ethics Statement

Ethical review and approval was not required for the animal study because No *in vivo* study was performed. The killing of the mice required for isolating the cells used in primary culture was approved by the commissions for Institutional Animal Care and Use at Saarland University, Saarland, Germany.

## Author Contributions

UB, JR, and ASt conceived the study and designed and interpreted the experiments. ASt performed and analyzed most of the experiments, performed the MS result analysis and interpretation. UB performed part of the experiments shown Figure 6 and all of Figure 7. OR conceived the CAPS1/2 chimera. HB and ASh performed the experiments shown in Figure 2. AB and ML performed part of the analysis shown in Figure 6. CF-T and VF performed MS measurements and initial data analysis. AH generated the CA-ATTO590. MK generated the lentiviruses. AHS and ML shared essential expertise. UB and JR supervised the study. UB, JR, and ASt wrote the manuscript with contributions from all authors. All the authors contributed to the article and approved the submitted version.

## Conflict of Interest

The authors declare that the research was conducted in the absence of any commercial or financial relationships that could be construed as a potential conflict of interest.

## Publisher’s Note

All claims expressed in this article are solely those of the authors and do not necessarily represent those of their affiliated organizations, or those of the publisher, the editors and the reviewers. Any product that may be evaluated in this article, or claim that may be made by its manufacturer, is not guaranteed or endorsed by the publisher.

## Footnotes

1
http://smart.embl-heidelberg.de/

2
http://pfam.xfam.org/

3
https://alphafold.ebi.ac.uk

## Abbreviations

CAPS: Calcium-dependent activator protein for secretion
CA-ATTO590: chloralkane-ATTO590
DIV: day *in vitro*
CAPS dKO: deletion of CAPS1 and CAPS2
DCV: dense core vesicle
DRG: dorsal root ganglion
IPs: immunoprecipitations
LDCVs: large dense core vesicles
MS: mass spectrometry
MHD1: Munc13 homology domain 1
PBS: phosphate-buffered saline
PSF: point spread functions
ROI: region of interest
SFV: Semliki-Forest virus
SC: spinal cord
STED: stimulated emission depletion
SVs: synaptic vesicles
SypHy: synaptophysin-pHluorin
WT: wild-type.
