# Supplementary material for: Localization of the Priming Factors CAPS1 and CAPS2 in Mouse Sensory Neurons Is Determined by Their N-Termini
Source: Front Mol Neurosci. 2022 Apr 14;15:674243. doi: 10.3389/fnmol.2022.674243 (PMC9049930; doi:10.3389/fnmol.2022.674243)

Localization of the priming factors CAPS1 and CAPS2 in mouse sensory neurons is determined by their N-termini

Supplementary Material

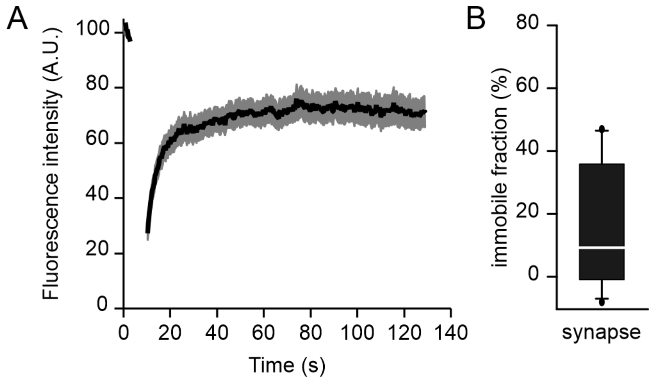

Supplementary Figure 1: mRFP fluorescence recovery after bleaching is much faster and complete than any CAPS protein. WT DRG neurons were transfected with a lentivirus encoding for mRFP before being co-cultured with WT SC neurons. After 9 DIV in culture, active synapses were stained with Syt1-C2 and FRAP experiment was performed as in Figure 7. **(A)** Average fluorescence recovery of the mRFP at synapses over time. Data are mean ± SEM. **(B)** Immobile CAPS fraction displayed as box plot. $N_{experiment}$ = 1, $n_{synapses}$ = 11.

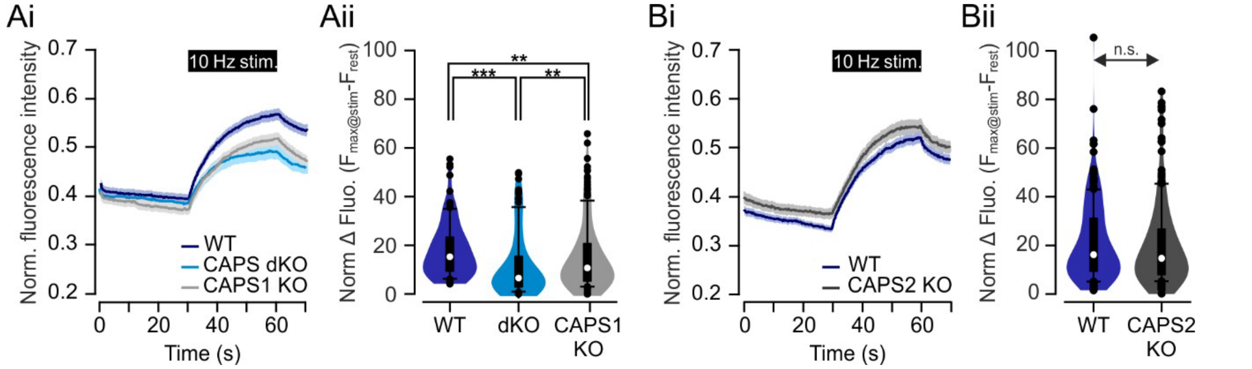

Supplementary Figure 2 CAPS1 but not CAPS2 deletion impairs synaptic transmission. (A) CAPS dKO and CAPS1 KO effect on synaptic transmission in DRG neurons. WT, CAPS dKO, or CAPS1 KO DRG neurons were isolated from E18/19 mice, infected with SypHy and co-cultured with WT SC neurons. **(Ai)** Normalized average SypHy signal at synapses in response to 10 Hz field electrode stimulation are shown for WT (blue), CAPS dKO (light blue), and CAPS1 KO neurons (light grey). Data are mean ± SEM. **(Aii)** Violin plot of the maximum normalized fluorescence intensity increase in SypHy elicited during 10 Hz stimulation. Experiments were performed on a minimum of three independent cultures for every genotype and the number of measured synapses was 119, 143, and 171 for WT, CAPS dKO, and CAPS1 KO neurons, respectively. **$p < 0.01$ or *** $p < 0.001$ ANOVA on rank with Dunn's post-hoc test. **(B)** CAPS2 KO effect on synaptic transmission in DRG neurons. WT or CAPS2 KO DRG neurons were isolated from P6 to P8 mice, infected with SypHy and co-cultured with WT SC neurons. **(Bi)** Normalized average SypHy fluorescence intensity measured at synapses in response to electrical stimulation for WT neurons (blue) and CAPS2 KO neurons (dark grey). Data are mean ± SEM. **(Bii)** Violin plot of the maximum normalized fluorescence intensity increase in SypHy elicited by 10 Hz electrical stimulation. Experiments were performed on a minimum of three independent cultures for every genotype. 241 and 184 synapses were measured in WT and CAPS2 KO neurons, respectively. n.s. = not significant.

Supplementary Figure 3 CAPS1 proteins are detected after IP by nano-LC-MS/MS.

(A) Protein sequence of mouse CAPS1 (SwissProt: Q80TJ1) is shown in black. Amino acids identified by MS/MS fragmentation are shown in red, covering 54% of the protein sequence. **(B)** Example of collision induced damage (CID) MS/MS fragmentation spectra (blue; y-ions, red; b-ions, green; +-NH3,+-H2O,+2H, parent) derived from the underlined tryptic mouse CAPS1 peptide in A analyzed by nano-LC ESI-MS/MS

A

CAPS1

```
1    MLDPSSSEEE SDEILEEERG KDVLGSAASG ARLSPSRTSE GSAGSAGMGG
51   SGAGAGVGAG GGGGSGASSG GGAGGLQPSS RAGGGRPSSP SPSVVSEKEK
101  EELERLQKEE EERKKRLQLY VFVMRCIAYP FNAKQPTDMA RROOKISKOO
151  LOTVKDRFOA FLNGETOIVA DEAFMNAVOS YYEVFLKSDR VARMVQSGGC
201  SANDSREVFK KHIEKRVRSL PEIDGLSKET VLSSWMAKFD AIYRGEEDPR
251  KQQARMTASA ASELILSKEQ LYEMFQNILG IKKFEHQLLY NACQLDNPDE
301  QAAQIRRELD GRLQMADQIA RERKFPKFVS KEMENMYIEE LKSSVNLLMA
351  NLESMPVSKG GEFKLQKLKR SHNASIIDMG EESENQLSKS DVLLSFSLEV
401  VIMEVOGLKS LAPNRIVYCT MEVEGGEKLQ TDQAEASKPT WGTQGDFSTT
451  HALPAVKVKL FTESTGVLAL EDKELGRVIL HPTPNSPKOS EWHKMTVSKN
501  CPDQDLKIKL AVRMDKPQNM KHSGYLWTIG KNVWKRWKKR FFVLVOVSOY
551  TFAMCSYREK KAEPQELLQL DGYTVDYTDP QPGLEGGRAF FNAVKEGDTV
601  IFASDDEQDR ILWVQAMYRA TGQSHKPVPP TQVQKLNAKG GNVPOLDAPI
651  SQFSGLKDAD RAQKHGMDEF ISSNPCNFDH ASLFEMVQRL TLDHRLNDSY
701  SCLGWFSPGO VFVLDEYCAR NGVRGCHRHL CYLRDLLERA ENGAMIDPTL
751  LHYSFAFCAS HVHGNRPDGI GTVTVEEKER FEEIKERLRV LLENQITHFR
801  YCFPFGRPEG ALKATLSLLE RVLMKDIVTP VPQEEVKTVI RKCLEQAALV
851  NYSRLSEYAK IEENQKDAEN VGRLITPAKK LEDTIRLAEL VIEVLQONEE
901  HHAEPHVDKG EAFAWWSDLM VEHAETFLSL FAVDMDAALE VOPPDTWDSF
951  PLFQLLNDFL RTDYNLCNGK FHKHLQDLFA PLVVRYVDLM ESSIAQSIHR
1001 GFERESWEPV KSLTSNLPNV NLPNVNLPKV PNLPVNIPLG IPOMPTFSAP
1051 SWMAAIYDAD NGSGTSEDLF WKLDALQTFI RDLHWPEEEF GKHLEQRLKL
1101 MASDMIESCV KRTRIAFEVK LOKTSRSTDF RVPQSICTMF NVMVDAKAOS
1151 TKLCSMEMGQ EHQYHSKIDE LIEETVKEMI TLLVAKFVTI LEGVLAKLSR
1201 YDEGTLFSSF LSFTVKAASK YVDVPKPGMD VADAYVTFVR HSQDVLRDKV
1251 NEEMYIERLF DQWYNSSMNI ICTWLTDRMD LQLHIYQLKT LIRMVKKTYR
1301 DFRLQGVLDS TLNSKTYETI RNRLTVEEAT ASVSEGGGLO GISMKDSDEE
1351 DEEDD
```

B

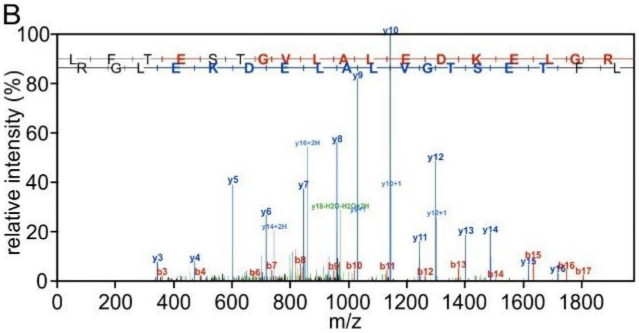

Supplement: Supplementary file 1 [file Data_Sheet_1.pdf]
